# Patient-specific variants of NFU1/NFU-1 disrupt cholinergic signaling in a model of multiple mitochondrial dysfunctions syndrome 1

**DOI:** 10.1242/dmm.049594

**Published:** 2023-02-01

**Authors:** Peter A. Kropp, Philippa Rogers, Sydney E. Kelly, Rebecca McWhirter, Willow D. Goff, Ian M. Levitan, David M. Miller, Andy Golden

**Affiliations:** ^1^ Laboratory of Biochemistry and Genetics, National Institute of Diabetes and Digestive and Kidney Diseases, National Institutes of Health, Bethesda, MD 20892, USA; ^2^ Biology Department, Kenyon College, Gambier, OH 43022, USA; ^3^ Department of Cell and Developmental Biology, Vanderbilt University, Nashville, TN 37235, USA; ^4^ Biology Department, Colgate University, Hamilton, NY 13346, USA; ^5^ Neuroscience Graduate Program, Vanderbilt University, Nashville, TN 37235, USA

**Keywords:** *C. elegans*, MMDS1, Acetylcholine, GABA, Motility, Mitochondria

## Abstract

Neuromuscular dysfunction is a common feature of mitochondrial diseases and frequently presents as ataxia, spasticity and/or dystonia, all of which can severely impact individuals with mitochondrial diseases. Dystonia is one of the most common symptoms of multiple mitochondrial dysfunctions syndrome 1 (MMDS1), a disease associated with mutations in the causative gene (*NFU1*) that impair iron–sulfur cluster biogenesis. We have generated *Caenorhabditis elegans* strains that recreated patient-specific point variants in the *C. elegans* ortholog (*nfu-1*) that result in allele-specific dysfunction. Each of these mutants, Gly147Arg and Gly166Cys, have altered acetylcholine signaling at neuromuscular junctions, but opposite effects on activity and motility. We found that the Gly147Arg variant was hypersensitive to acetylcholine and that knockdown of acetylcholine release rescued nearly all neuromuscular phenotypes of this variant. In contrast, we found that the Gly166Cys variant caused predominantly postsynaptic acetylcholine hypersensitivity due to an unclear mechanism. These results are important for understanding the neuromuscular conditions of MMDS1 patients and potential avenues for therapeutic intervention.

## Teaser

**Summary:** Patient-specific variants in NFU1/NFU-1 affect motility and movement in *C. elegans* due to aberrant cholinergic signaling, but variants have different effects on presynaptic and postsynaptic function.

## INTRODUCTION

Multiple mitochondrial dysfunctions syndrome 1 (MMDS1) is a rare autosomal-recessive disorder caused by mutations in the protein NFU1. NFU1 is responsible for trafficking iron–sulfur clusters (ISCs) to recipient proteins within mitochondria that require ISCs for function (reviewed in Braymer and Lill, 2017; Maio et al., 2020; Rouault and Maio, 2017). Disruptions of ISC biogenesis or delivery result in significant negative effects on mitochondrial function, including impaired oxidative respiration and excessive oxidative stress (Maio and Rouault, 2020; Stehling and Lill, 2013; Vanlander and Coster, 2018; Wachnowsky et al., 2018). Consequently, pathogenic mutations of NFU1 cause severe disease. Tragically, MMDS1 is pediatric lethal in nearly every known case (Ahting et al., 2015; Ames et al., 2020; Bai and Kong, 2017; Birjiniuk et al., 2020; Cameron et al., 2011; Ferrer-Cortès et al., 2016; Invernizzi et al., 2014; Jin et al., 2017; Navarro-Sastre et al., 2011; Nizon et al., 2014; Seyda et al., 2001; Souza et al., 2018; Tonduti et al., 2015; Uzunhan et al., 2020).

Complicating our understanding of MMDS1 pathogenesis are the small number of cases (38 reported to date) and the diverse genetic variations of *NFU1* that are potentially pathogenic. Twenty different *NFU1* variants, including point mutations, frameshifts and small deletions, have been reported. Further, the *NFU1* locus in approximately half of MMDS1 patients is compound heterozygous for different *NFU1* variants. Most of these *NFU1* alleles are thought to result in severe loss of NFU1 function. Consistent with this assumption, typical MMDS1 patient symptoms include neurological regression, reduced motor control (dystonia) and pulmonary hypertension (Ahting et al., 2015; Ames et al., 2020; Bai and Kong, 2017; Birjiniuk et al., 2020; Cameron et al., 2011; Ferrer-Cortès et al., 2016; Invernizzi et al., 2014; Jin et al., 2017; Navarro-Sastre et al., 2011; Neveu et al., 2022; Nizon et al., 2014; Seyda et al., 2001; Souza et al., 2018; Tonduti et al., 2015; Uzunhan et al., 2020). Neurological regression is likely to be caused by leukoencephalopathy, a lesion of white matter in the brain, and a common finding in reports of MMDS1 (Ames et al., 2020; Ferrer-Cortès et al., 2016; Invernizzi et al., 2014; Jin et al., 2017; Navarro-Sastre et al., 2011; Nizon et al., 2014; Souza et al., 2018; Tonduti et al., 2015). Pulmonary hypertension is likely to be caused by hyperproliferation of endothelial cells of the pulmonary vasculature (James et al., 2020; Niihori et al., 2020), but the underlying mechanism remains unclear. The mechanism underlying neuromuscular dysfunction in MMDS1 has not been directly explored.

Regulated control of movement depends on neuromuscular junctions, specialized synapses between motor neurons and skeletal muscle. Neurotransmitters secreted into this synapse drive either contraction or relaxation of the muscle. Skeletal muscle contraction is stimulated by the neurotransmitter acetylcholine (ACh). Cholinergic motor neurons secrete ACh into the neuromuscular junction, where it binds to either the fast-acting nicotinic ACh receptor (nAChR) or slower-acting muscarinic ACh receptor (mAChR). nAChRs are ligand-gated Na^+^ channels, whereas mAChRs are G-protein-coupled receptors that regulate second-messenger cascades (reviewed in Mukund and Subramaniam, 2020). The neurotransmitter γ-aminobutyric acid (GABA), secreted from GABAergic motor neurons, also acts at the neuromuscular junction, where it binds to the GABA receptor (GABAR) to antagonize muscle contraction. Like ACh receptors (AChRs), GABARs can be fast or slow acting. The fast-acting GABA_A_ receptor is also a ligand-gated ion channel and allows Cl^−^ to enter the muscle cell, thereby promoting muscle relaxation. The slow-acting GABA_B_ receptor relies on second-messenger cascades that also inhibit muscle contraction (Bettler et al., 2004; Ochoa-de la Paz et al., 2021; Shaye et al., 2021). Dysregulation of either of these signaling pathways can result in muscular phenotypes.

We previously established a *Caenorhabditis elegans* model of MMDS1 by recreating exact patient-specific NFU1 variants in the *C. elegans* ortholog NFU-1. We studied five variants affecting the ISC interaction domain of NFU1/NFU-1 and demonstrated that they caused dysregulation of cellular iron as well as oxidative stress (Kropp et al., 2021a). In agreement with biochemical studies from another research group (Wachnowsky et al., 2017; Wesley et al., 2017a,b), our data supported a role for altered NFU1/NFU-1 dimerization as the cause of allele-specific phenotypes of NFU-1 variants. Indeed, altered dimerization dynamics of NFU1/NFU-1 not only impairs ISC delivery, but can also expose ISCs to the intracellular environment, causing distinctive metallostress phenotypes. We thus hypothesized that these patient-specific variants would also result in unique neuromuscular phenotypes and could help elucidate the mechanism of neuromuscular dysfunction in MMDS1 patients. *C. elegans* has long been an excellent model organism for investigating neuromuscular function (Brenner, 1974), in no small part due to its exquisitely mapped neural connectome and gene expression atlas for the nervous system (Cook et al., 2019; Taylor et al., 2021) Both ACh and GABA signaling are well conserved at genetic and functional levels (Jones and Sattelle, 2004; Mongan et al., 1998; Kaupmann et al., 1997; Schofield et al., 1987), making the findings here relevant to human MMDS1.

In this study, we focused on two patient-specific NFU-1 variants: Gly147Arg and Gly166Cys. These two variants were chosen because (1) our previous work demonstrated that they have phenotypes that are divergent from a full loss of NFU-1 function, and (2) they are amongst the most common variants observed in both homozygous and compound heterozygous cases of MMDS1. These two alleles account for >50% of homozygous MMDS1 cases. We found that Gly147Arg and Gly166Cys result in allele-specific phenotypes, including dysregulation of ACh signaling, that differ from both wild type (WT), a full *nfu-1* deletion (*nfu-1*Δ) and each other. Dysregulation does not appear to be caused by oxidative stress, but rather hypersecretion of ACh by Gly147Arg motor neurons in an apparent phenotypic gain-of-function and hypersensitivity to ACh of Gly166Cys muscle cells. Re-expression of WT *nfu-1* in muscles was capable of rescuing motility phenotypes in both Gly147Arg as well as Gly166Cys, whereas rescue by re-expression of *nfu-1* in neurons was more variable in both variants. Motility phenotypes of Gly147Arg were rescued by reducing secretion of ACh, indicating a potential avenue for therapeutic intervention in MMDS1 patients with this specific variant.

## RESULTS

### *nfu-1* mutants have motility phenotypes on solid medium

We previously demonstrated that patient-specific MMDS1 variants in the *C. elegans* gene *nfu-1* display mitochondrial stress, including morphological and functional impairments, in multiple tissues (Kropp et al., 2021a). Because mitochondrial stress can result in movement defects due to dysfunction in the neuromuscular system, we assessed the motility of *nfu-1* variants. We focused on the NFU-1 variants Gly147Arg and Gly166Cys (for clarity, these variants will be referenced by the corresponding amino acid change). The Gly147Arg and Gly166Cys variants were compared to the wild type (WT) and to a genetic deletion of *nfu-1* (*nfu-1*Δ). Because Gly166Cys and *nfu-1*Δ arrest during late larval development (∼L4 larval stage), all analyses were performed at this stage regardless of genotype.

**Fig. 1.**
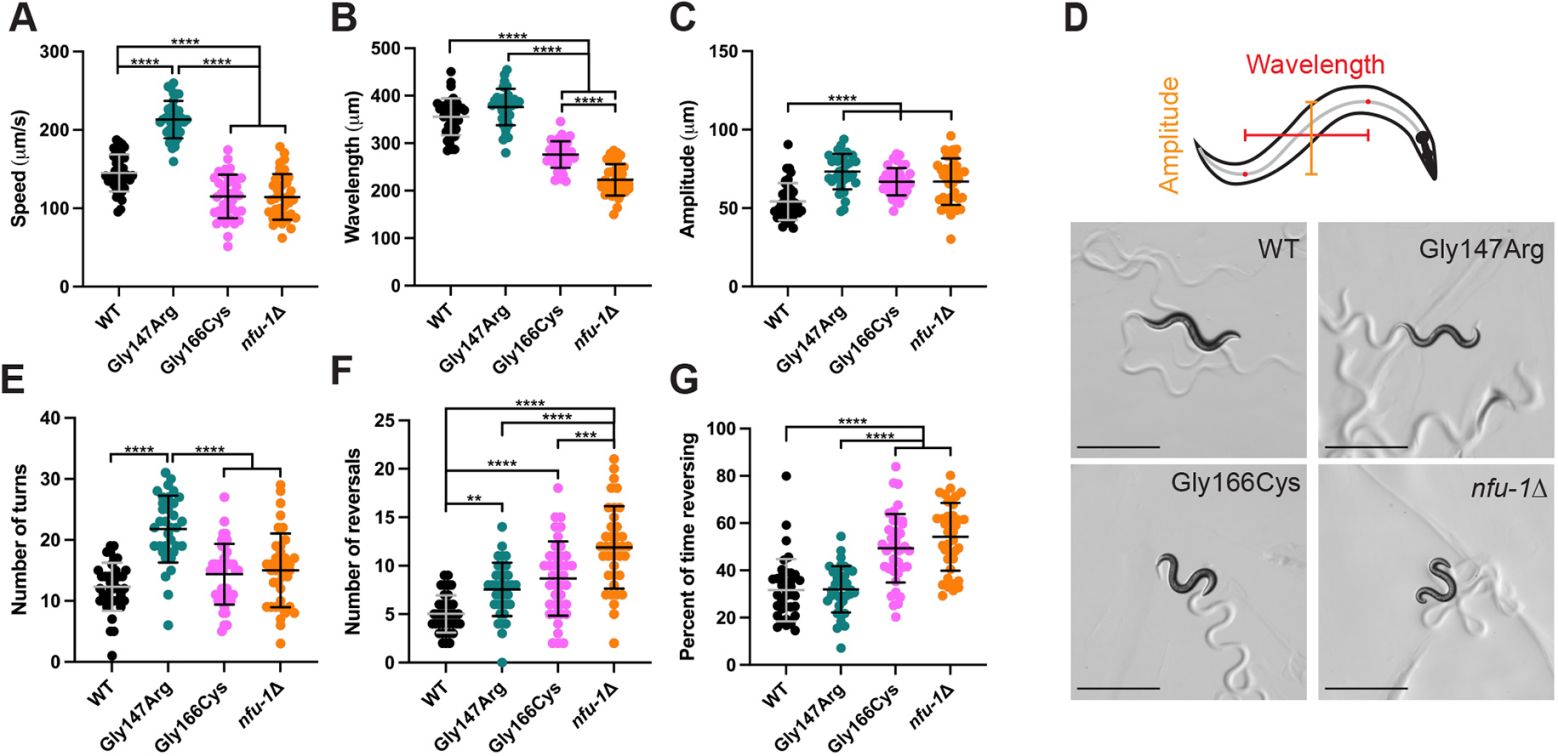
**Crawling defects of *nfu-1* variants on solid medium.** (A) Speed of crawling. (B) Wavelength of sinusoidal wave. (C) Amplitude of sinusoidal wave. (D) Schematic of wavelength and amplitude, and representative images of animals analyzed. (E) Number of turns in 1 min. (F) Number of reversals in 1 min. (G) Percentage of time reversing. Each data point is an individual animal (*n*=36-40). **P≤0.01; ***P≤0.001; ****P≤0.0001 by one-way ANOVA with Tukey correction for multiple comparisons. Scale bars: 500 µm. WT, wild type.

We used video recordings to quantify the motility of *nfu-1* variants on solid medium. Videos of animals crawling on an agarose pad were captured and analyzed with the WormLab system (Restif et al., 2014). Gly166Cys and *nfu-1*Δ moved (either forward or backward) at significantly slower speeds than WT; however, Gly147Arg moved significantly faster than all other groups (Fig. 1A). In *C. elegans*, forward movement depends on a sinusoidal wave that begins at the nose and transits through the body to the tail. Aberrations in this waveform can be indicative of altered neuromuscular function. Both Gly166Cys and *nfu-1*Δ showed a decreased wavelength for sinusoidal movement in comparison to WT (Fig. 1B,D) that is indicative of a more compact wave. This phenotype was most severe for *nfu-1*Δ. All *nfu-1* variants – Gly147Arg, Gly166Cys and *nfu-1*Δ – showed an increased wave amplitude (Fig. 1C,D), indicating deeper body bends.

We also analyzed turning and reversals, body movements normally observed during foraging behavior as animals search for food. Turning occurs when animals stop and change direction before continuing forward movement. Reversals occur when animals stop forward movement and instead move backward by initiating sinusoidal waves at the tail rather than at the nose. Gly147Arg turned more frequently than any other group, but no difference was observed between WT and Gly166Cys and *nfu-1*Δ (Fig. 1E). Further, each *nfu-1* variant reversed more frequently than WT, with *nfu-1*Δ switching from forward to reverse movement more than any other group (Fig. 1F). This increased incidence of reversals resulted in Gly166Cys and *nfu-1*Δ spending more than 50% of the time reversing, significantly more than either WT or the Gly147Arg mutant (Fig. 1G). Despite an increased incidence of reversals, Gly147Arg did not spend more time reversing than WT, thus the increased reversal incidence could be a function of more rapid movement overall. Additionally, the decreased speeds of Gly166Cys and *nfu-1*Δ were not a result of the time reversing as speed was calculated from movement in both directions (Fig. 1A). Together, these data demonstrate that all *nfu-1* variants show motility defects on solid medium, with the Gly147Arg variant displaying hyperactive behavior (e.g. more frequent turns, faster speed), whereas the Gly166Cys variant and *nfu-1*Δ were hypoactive (e.g. less frequent turns, slower speed). All variants showed alterations in body waveform (e.g. decreased wavelength and amplitude). Together, these changes suggested that neuromuscular function was disrupted in Gly147Arg, Gly166Cys and *nfu-1*Δ.

### Swimming defects in *nfu-1* variants

**Fig. 2.**
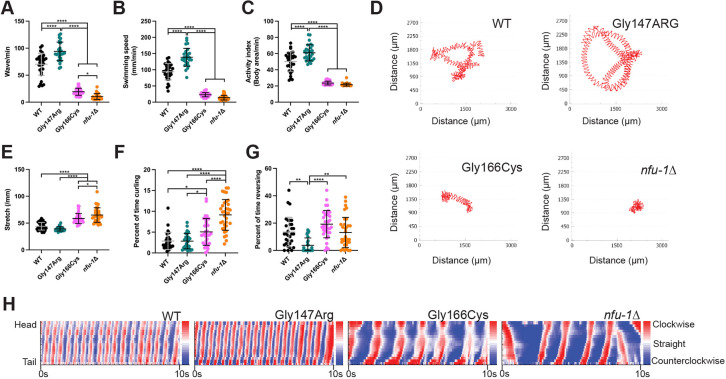
**Swimming defect of *nfu-1* variants in liquid.** (A) Wave rate. (B) Swimming speed. (C) Activity index. (D) Representative traces of animal center point in 1 min. (E) Stretch: a measure of bend in the body during a wave where stretch is measured as the inverse of distance (i.e. more stretch brings the anterior and posterior of the animal together giving a larger value). (F) Percentage of time curling. (G) Percentage of time reversing. (H) Representative curvature maps of individual animals during measurement. Color represents the body's curvature during a wave, with deeper red indicating a deeper clockwise curve and deeper blue indicating a deeper counterclockwise curve. In each frame (*x*-axis point), curvature was measured across the length of each animal in 17 slices (*y*-axis point). Each data point is an individual animal (*n*=28-35). *P≤0.05; **P≤0.01; ****P≤0.0001 by one-way ANOVA with Tukey correction for multiple comparisons.

As an additional test of potential motility defects in *nfu-1* variants, we analyzed swimming behavior. *C. elegans* placed in liquid will characteristically thrash back and forth in a sinusoidal movement called swimming. We captured videos of animals and used WormLab to analyze swimming. In agreement with the locomotor phenotypes observed on solid medium, Gly147Arg showed hyperactive movement (elevated wave initiation rate), whereas the Gly166Cys variant and *nfu-1*Δ were severely hypoactive compared to WT (reduced wave initiation rate) (Fig. 2A; Movies 1-4). These allele-specific effects were also observed for swimming speed and overall activity (Fig. 2B-D). Similar to the decreased wavelength on solid medium (Fig. 1B), Gly166Cys and *nfu-1*Δ showed significantly more stretch to individual waves (closeness of head and tail; Fig. 2E,H), indicating deeper curves of individual body waves. This effect was slightly greater in *nfu-1*Δ than in Gly166Cys. Yet, both Gly166Cys and *nfu-1*Δ spent significantly more time curling (Fig. 2F), a behavior that occurs normally but is exaggerated in these variants; *nfu-1*Δ demonstrated this phenotype most severely. As also observed for crawling on solid medium, *C. elegans* will occasionally reverse while swimming, with waveforms beginning at the tail rather than at the nose. This behavior is apparent as a change in the direction of body bend while swimming (see *nfu-1*Δ in Fig. 2H). Surprisingly, the Gly147Arg variant spent significantly less time reversing than any other group, whereas this difference was not observed for Gly147Arg on solid medium (Fig. 2G). The significance of this disparity is unclear, but may be attributable to distinct differences in the operation of movement circuits in crawling versus swimming behavior (Pierce-Shimomura et al., 2008; Vidal-Gadea et al., 2011). Collectively, the crawling and swimming phenotypes of these variants indicate that the Gly147Arg variant causes a gain-of-function in locomotion, whereas both the Gly166Cys variant and *nfu-1*Δ cause loss-of-function in locomotion. Importantly, the gain-of-function observed in Gly147Arg is exclusively a phenotypic effect as this allele is not genetically dominant (Fig. S1). Thus, the Gly147Arg variant is recessive but presents with a gain-of-function locomotion phenotype.

### Neuromuscular signaling is disrupted in all *nfu-1* variants

**Fig. 3.**
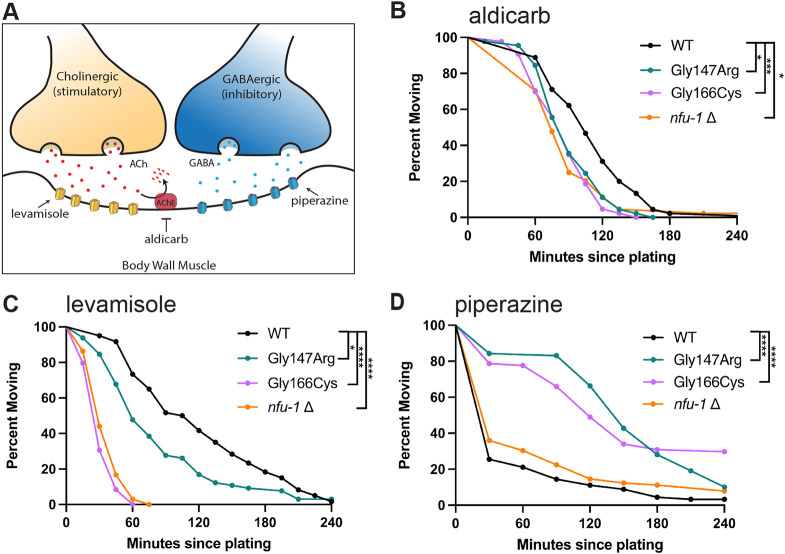
***nfu-1* variants experience different sensitivities to neuromuscular signaling pathways.** (A) Schematic of a simplified neuromuscular junction. Cholinergic neurons stimulate body wall muscle contraction by secreting acetylcholine (ACh; red dots), which binds to the ACh receptor (AChR; shown as yellow/orange receptor). ACh is degraded in the synapse by acetylcholinesterase (AChE). GABAergic neurons inhibit body wall muscle contraction by secreting GABA (blue dots), which binds to the GABA receptor (GABAR; blue receptor). Drugs used: levamisole, an AChR agonist; aldicarb, an AChE antagonist; and piperazine, a GABAR agonist. (B) Aldicarb paralysis curve (*n*=75-105). (C) Levamisole paralysis curve (*n*=45-65). (D) Piperazine paralysis curve (*n*=89-95). Data plotted as Kaplan–Meier survival curves. *P≤0.05; ***P≤0.001; ****P≤0.0001 by log-rank analysis with Bonferroni correction for multiple comparisons.

Given the motility defects observed in the *nfu-1* variants, function of the neuromuscular junction was next assessed. In both *C. elegans* body wall muscle and human skeletal muscle, contraction is stimulated by ACh release from cholinergic motor neurons (Fig. 3A). Relaxation is induced by secretion of GABA from GABAergic motor neurons (Fig. 3A). The coordinated action of these neurotransmitters is essential for the contraction and relaxation cycles that drive the sinusoidal movement of *C. elegans* (Jorgensen and Nonet, 1995; Richmond and Jorgensen, 1999).

Neuromuscular signaling was first assessed by exposing animals to aldicarb, an inhibitor of acetylcholinesterase (AChE), which normally degrades ACh in the synapse (Fig. 3A). Treatment with aldicarb results in accumulation of ACh and paralysis due to hypercontraction (Mahoney et al., 2006). Surprisingly, all *nfu-1* variants were hypersensitive to aldicarb (Fig. 3B; Table S1). Hypersensitivity to aldicarb could be caused by either enhanced sensitivity to ACh or an attenuated response to GABA. To distinguish between these models, we performed paralysis assays with drugs that directly target either cholinergic or GABAergic signaling via their respective receptor. Levamisole is an nAChR agonist that activates muscle nAChRs and, like aldicarb, causes hypercontraction and paralysis. In agreement with the hypothesis that *nfu-1* variants are hypersensitive to ACh, each variant was also hypersensitive to levamisole, albeit to differing degrees (Fig. 3C; Table S1). Piperazine is an agonist of the GABA_A_ receptor, and thus exposure of *C. elegans* to this drug causes flaccid paralysis due to complete relaxation of the body wall muscles. Intriguingly, both Gly147Arg and Gly166Cys were resistant to piperazine, whereas *nfu-1*Δ was unaffected (Fig. 3D; Table S1). Together, these results suggested that both ACh and GABA signaling are dysfunctional in Gly147Arg and Gly166Cys. Further, these findings indicated that there must be a mechanistic difference between Gly166Cys and *nfu-1*Δ, at least with respect to GABA signaling.

### Loss of muscle *nfu-1* causes more severe motility defects than loss of neuronal *nfu-1*

We sought next to investigate whether neuromuscular signaling defects were caused by presynaptic (neuronal) or postsynaptic (muscular) effects. For example, hypersensitivity to ACh could be caused by elevated secretion of ACh, hypersensitivity of target nAChRs or a combination of both. Therefore, mutant *C. elegans* strains with either presynaptic or postsynaptic alterations in ACh and GABA signaling were analyzed with either aldicarb, levamisole or piperazine. The drug-induced paralysis curves with each of these mutants were then compared to that of *nfu-1* variants. Strains tested were *tom-1(ok285)*, *rig-3(ok2156)*, *unc-47(n2409)* and *unc-49(e407)*. TOM-1 is a syntaxin-binding protein that positively regulates ACh vesicular docking through interactions with syntaxins (Gracheva et al., 2006). RIG-3 is a cell-surface immunoglobulin-domain-containing protein that positively regulates the abundance of the ACR-16 subunit of muscle AChRs (Babu et al., 2011). UNC-47 is the vesicular GABA transporter and is essential for packaging of GABA into secretory vesicles (Vashlishan et al., 2008). UNC-49 is an essential GABA_A_ subunit of the GABAR (Weeks et al., 2018). These strains were generally hypersensitive to aldicarb and levamisole, as expected based on published data (Fig. S2, Table S2) (Babu et al., 2011; Bhardwaj et al., 2020; Gracheva et al., 2006; Vashlishan et al., 2008; Weeks et al., 2018). Only *tom-1(ok285)* and *unc-49(e407)* were resistant to piperazine (Fig. S2, Table S2). Because of the specificity of levamisole and piperazine to the receptor of their respective signaling pathway, we focused on these two drugs rather than aldicarb in further analyses. When comparing both levamisole and piperazine paralysis curves, the *tom-1(ok285)* and *unc-49(e407)* strains most closely resembled the *nfu-1* variants, with *tom-1(ok285)* more similar to Gly147Arg and *unc-49(e407)* more similar to Gly166Cys and *nfu-1*Δ. Because TOM-1 is a presynaptic protein and UNC-49 is a postsynaptic protein, these data provided the first indication that Gly147Arg may cause a presynaptic defect and that Gly166Cys and *nfu-1*Δ may disrupt postsynaptic function.

**Fig. 4.**
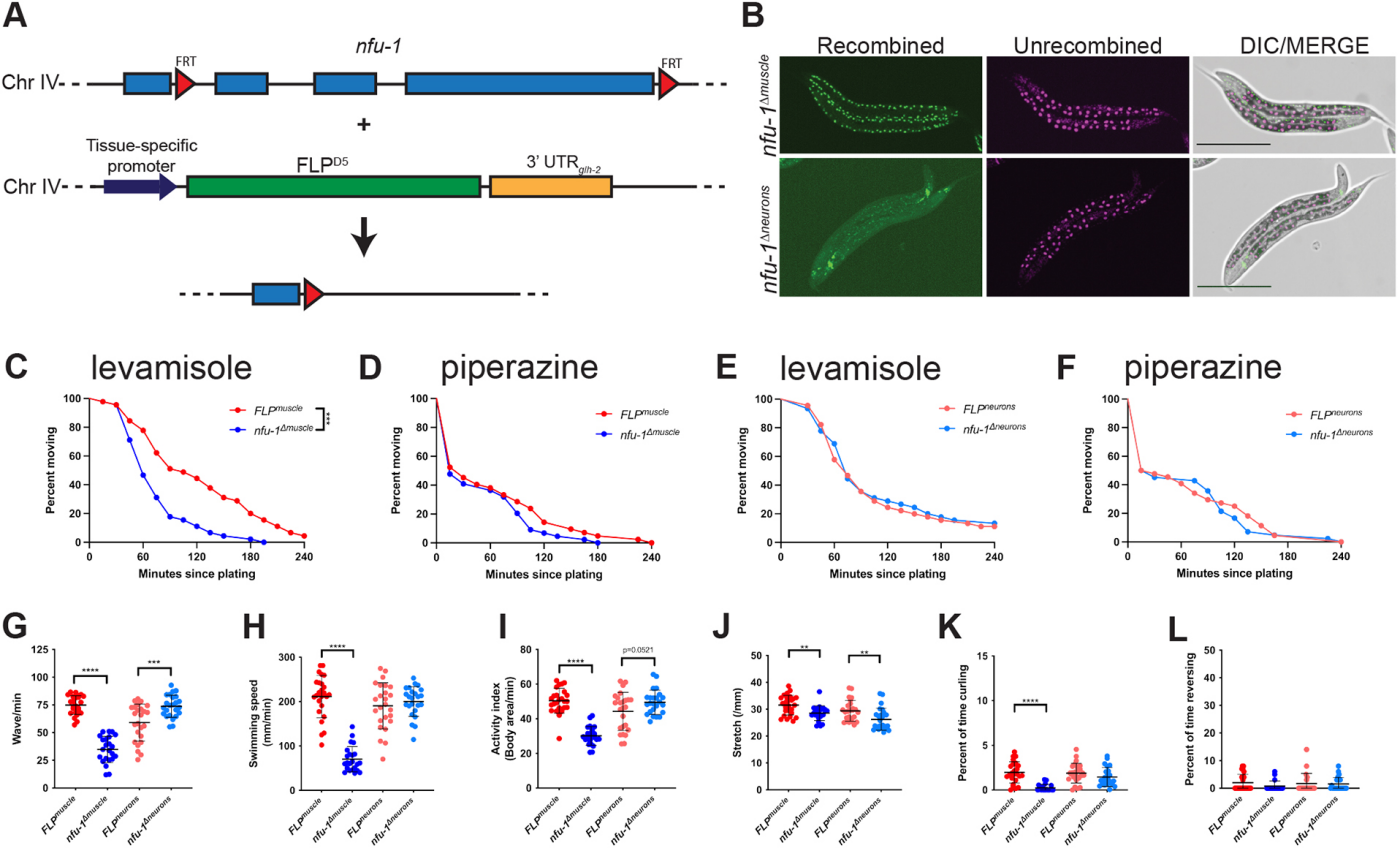
**Muscle-specific knockout of *nfu-1* results in more severe neuromuscular defects than neuron-specific knockout of *nfu-1*.** (A) Schematic of FLP/FRT system used for tissue-specific knockouts of *nfu-1*. All but the first exon of *nfu-1* was removed. Transgenic reporter construct shown in Fig. S3. Chr, chromosome; FLP, flippase; FRT, flippase recognition target. (B) Representative maximum-intensity projections of confocal images of *nfu-1^Δmuscle^* and *nfu-1^Δneurons^*. Cells in which recombination successfully occurred express GFP (green) and all other cells express mCherry (magenta). Scale bars: 250 µm. DIC, differential interference contrast. (C,E) Levamisole paralysis curve for muscle-specific (C) and neuron-specific (E) knockout of *nfu-1* (*n*=45). (D,F) Piperazine paralysis curve for muscle-specific (D) and neuron-specific (F) knockout of *nfu-1* (*n*=42-44). Data plotted as Kaplan–Meier survival curves. (G) Wave rate. (H) Swimming speed. (I) Activity index. (J) Stretch. (K) Percentage of time curling. (L) Percentage of time reversing. Each data point represents an individual animal (*n*=23-27). For C, ***P≤0.001 by log-rank analysis. For G-L, **P≤0.01; ***P≤0.001; ****P≤0.0001 by parallel two-tailed Student's *t*-tests.

To further investigate the presynaptic and postsynaptic roles of *nfu-1*, neuron-specific and muscle-specific *nfu-1* knockouts were generated using a flippase (FLP)/flippase recognition target (FRT) system (Muñoz-Jiménez et al., 2017). Using CRISPR insertions, the endogenous *nfu-1* locus was flanked by FRT sites (Fig. 4A). When combined with strains expressing FLP under control of a tissue-specific promotor, *nfu-1* was excised early in development in those cells in which FLP was expressed. The strains generated were the body-wall-specific knockout AG618 [*nfu-1(av246)*; *bqSi294; bqSi495*] and the neuron-specific knockout AG621 [*nfu-1(av246)*; *bqSi294; bqSi506*]. For simplicity, these strains will be referred to as *nfu-1^Δmuscle^* and as *nfu-1^Δneurons^*, respectively. The FLP-expressing parent strains [BN503 (*bqSi294; bqSi495*) and BN507 (*bqSi294; bqSi506*), respectively] were included as controls; these strains will be referred to as *FLP^muscle^* and *FLP^neurons^*, respectively. All four strains were healthy with no obvious growth defects.

To confirm efficacy of the FLP, imaging of an internal fluorescent reporter and gene expression analysis were performed. The FLP-expressing strains all contained an internal transgenic reporter of recombination (Fig. S3A) (Muñoz-Jiménez et al., 2017). This reporter contains the *mCherry* sequence including stop codon flanked by FRT sites and the *GFP* sequence in that order. Cells that do not express FLP express mCherry but not GFP. Cells that express the FLP express GFP but not mCherry because the *mCherry* sequence and stop codon have been removed. Upon confocal imaging of the *nfu-1^Δmuscle^* and *nfu-1^Δneurons^* animals, GFP was observed in body wall muscle cells of *nfu-1^Δmuscle^* and in neurons of *nfu-1^Δneurons^*, as expected (Fig. 4B). To confirm *nfu-1* excision, we used fluorescence-activated cell sorting (FACS) to isolate GFP^+^ cells (Fig. S3B,C) for mRNA extraction and measured *nfu-1* expression by quantitative reverse transcriptase PCR (qRT-PCR). Because FLP is active in GFP^+^ cells, *nfu-1* mRNA should be reduced in GFP^+^ cells from either *nfu-1^Δmuscle^* or *nfu-1^Δneurons^* animals. As expected, *nfu-1* expression was undetectable in the body wall muscle (GFP^+^) cells of *nfu-1^Δmuscle^* and undetectable in neurons (GFP^+^) of *nfu-1^Δneurons^*. Additionally, *nfu-1* expression was robust in all other (mCherry^+^) cells from each sample (Table S3). Therefore, we conclude that the FLP/FRT system efficiently excised *nfu-1* in a tissue-specific manner.

The tissue-specific *nfu-1* knockouts were subjected to levamisole and piperazine paralysis assays at the L4 larval stage as previously conducted for the *nfu-1* variants. Intriguingly, *nfu-1^Δmuscle^* was hypersensitive to levamisole but showed no change in sensitivity to piperazine, indicating that *nfu-1* is required for normal ACh signaling in body wall muscle, but dispensable for normal GABA signaling (Fig. 4C,D; Table S4). Surprisingly, *nfu-1^Δneuron^* showed no alterations in sensitivity to either levamisole or piperazine (Fig. 4E,F; Table S4), suggesting that *nfu-1* is dispensable for motor neuron function in *C. elegans*. To confirm that motor neurons were included in the recombined cells of the *nfu-1^Δneuron^* strain, we used qRT-PCR to measure expression of motor neuron-specific genes (*unc-17*, *unc-3*, *cha-1*, *cho-1*) in the GFP^+^ and mCherry^+^ samples from sorted *nfu-1^Δneurons^* cells. Each of these genes was highly enriched in the GFP^+^ cells compared to the mCherry^+^ cells, indicating that the FLP was indeed effective in motor neurons (Table S4).

Swimming characteristics of the tissue-specific *nfu-1* knockouts were also assessed. As with all other assays, analysis was initially performed at the L4 stage. Most swimming phenotypes observed in the *nfu-1* variants were either absent or subtle at the L4 stage of the tissue-specific knockouts (Fig. S3D-I). However, we observed during routine culture that *nfu-1^Δmuscle^* animals became progressively more uncoordinated with age. To quantify this effect, we analyzed the movement of day 1 adult (D1A) animals for swimming phenotypes. At D1A, many phenotypes were substantially more pronounced. The wave initiation rate was significantly reduced in *nfu-1^Δmuscle^* and slightly, but significantly, elevated in *nfu-1^Δneurons^* (Fig. 4G). Swimming speed and the activity index were likewise reduced in *nfu-1^Δmuscle^* (Fig. 4H,I), yet these two phenotypes did not reach statistical significance in *nfu-1^Δneurons^*. The stretch phenotype was slightly but significantly reduced in *nfu-1^Δmuscle^* and *nfu-1^Δneurons^* (Fig. 4J). Curling was significantly reduced in *nfu-1^Δmuscle^*, with no change in *nfu-1^Δneurons^*, whereas reverse swimming remained unchanged (Fig. 4K,L). These data show that the hypoactivity of *nfu-1^Δmuscle^* correlates with results obtained with Gly166Cys and *nfu-1*Δ (Fig. 2), whereas the subtle hyperactivity phenotypes of *nfu-1^Δneurons^* mirror our findings with Gly147Arg (Fig. 2). Collectively, the tissue-specific *nfu-1* knockouts demonstrate that the necessity for *nfu-1* is different in motor neurons and muscle cells, and that loss of *nfu-1* activity in either cell type can produce unique movement phenotypes.

### Re-expression of *nfu-1* partially rescues many motility phenotypes

**Fig. 5.**
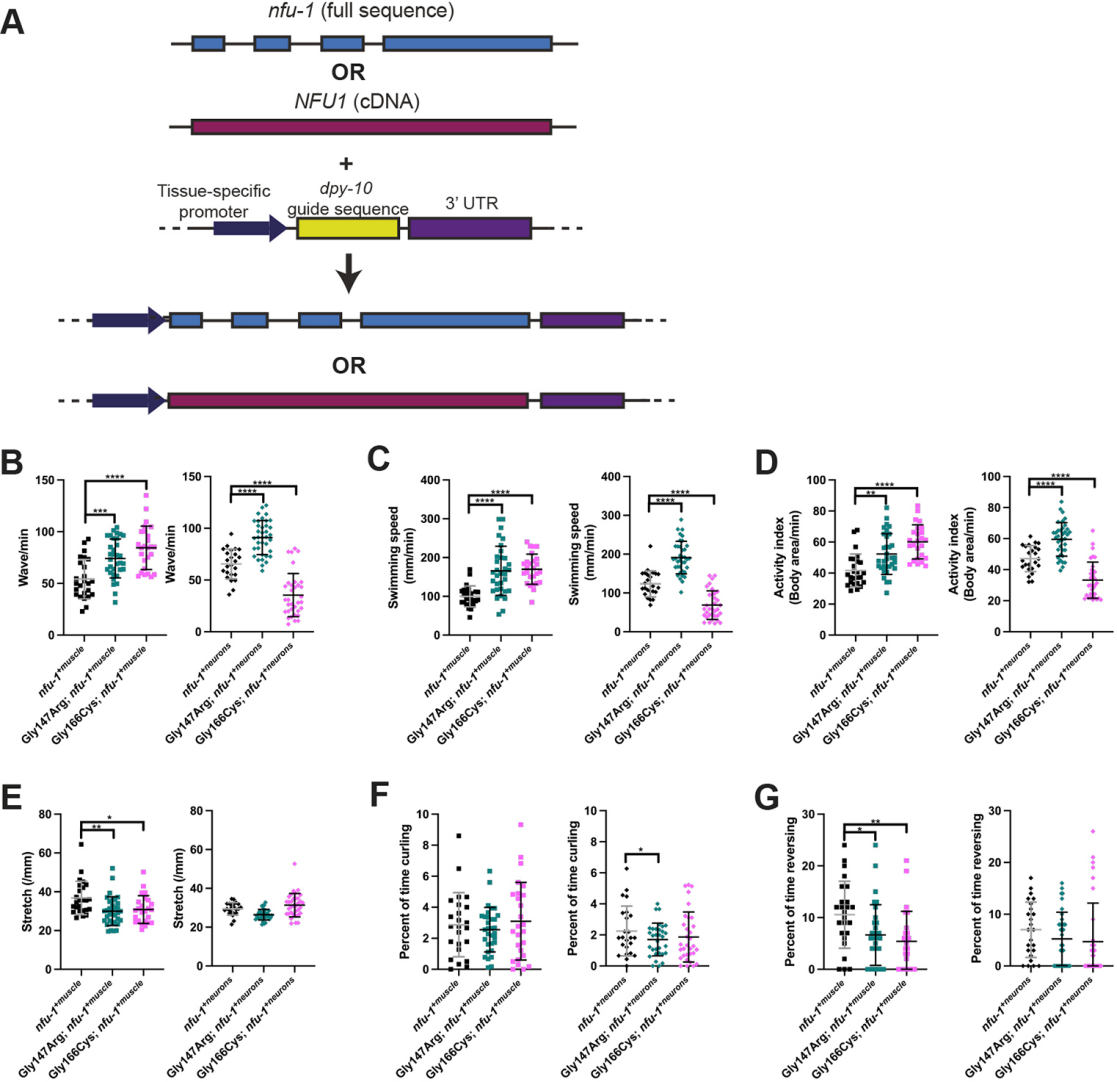
**Tissue-specific re-expression of *nfu-1* alone and in patient-specific variants.** (A) Schematic representation of the SKI LODGE approach for single-copy transgene insertion. (B) Wave rate. (C) Swimming speed. (D) Activity index. (E) Stretch. (F) Percentage of time curling. (G) Percentage of time reversing. Each data point represents an individual animal (*n*=25-37). For B-G, *P≤0.05; **P≤0.01; ***P≤0.001; ****P≤0.0001 from WT by one-way ANOVA with Dunnett correction for multiple comparisons.

In a complementary approach to the tissue-specific knockouts, we transgenically re-expressed the WT *nfu-1* sequence with tissue-specific promoters using the SKI LODGE system of single-copy transgene insertions (Fig. 5A) (Silva-García et al., 2019). As a proof of principle, we engineered both *C. elegans nfu-1* (*avIs286 [eft-3p::nfu-1::unc-54 3′UTR] V*) and human *NFU1* (*avIs287 [eft-3p::NFU1::unc-54 3′UTR] V*) for expression in all somatic cells (referred to as *nfu-1^+soma^* and *NFU1^+soma^*, respectively). Each of these strains was healthy and had largely normal motility, although slight but significant differences from WT in swimming speed (slightly increased in most transgenic lines) and stretch (slightly decreased in most transgenic lines) were observed (Table 1). We crossed these alleles into *nfu-1*Δ (*nfu-1*Δ; *nfu-1^+soma^* and *nfu-1*Δ; *NFU1^+soma^*) to determine whether each was capable of restoring normal motility. All motility phenotypes were significantly improved in *nfu-1*Δ; *nfu-1^+soma^*, with every measure being rescued to near-WT levels (Table 1; Fig. S4). Although marginally less effective than *nfu-1^+soma^*, motility phenotypes were partially rescued in *nfu-1*Δ; *NFU1^+soma^* (Table 1; Fig. S4), confirming that NFU-1 is the functional ortholog of NFU1, but human NFU1 may not be fully functional when expressed in *C. elegans*. These results indicated that this transgenic system was viable for tissue-specific *nfu-1* rescue experiments.

**Table 1.**
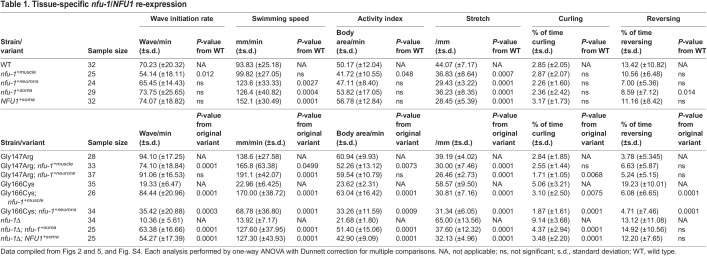
Tissue-specific *nfu-1*/*NFU1* re-expression

We next generated WT *nfu-1* re-expression constructs to restore expression in neurons (*avIs285 [rab-3p::nfu-1::rab-3 3′ UTR] V*) and muscles (*avIs284 [myo-3p::nfu-1::unc-54 3′ UTR] I*) (referred to as *nfu-1^+neurons^* and *nfu-1^+muscle^*, respectively). These transgenic lines were healthy, but *nfu-1^+muscle^* did have a slight reduction in wave initiation rate, and both lines had decreased stretch, similar to the somatic re-expression lines (Table 1). These two transgenes were crossed into the Gly147Arg and Gly166Cys variants, yielding the strains Gly147Arg; *nfu-1^+neurons^*, Gly147Arg; *nfu-1^+muscle^*, Gly166Cys; *nfu-1^+neurons^* and Gly166Cys; *nfu-1^+muscle^*. Swimming behavior of these strains was analyzed with WormLab.

Based upon the tissue-specific knockouts, we expected *nfu-1^+neurons^* to more effectively rescue Gly147Arg than *nfu-1^+muscle^*. However, re-expression of WT *nfu-1* had a greater effect when expressed in muscles of Gly147Arg animals than in neurons (Fig. 5B-G, Table 1). Most notably, the wave initiation rate and activity index were restored to near-WT levels in Gly147Arg; *nfu-1^+muscle^* (Table 1). There was no rescue of the reversal phenotype of Gly147Arg in Gly147Arg; *nfu-1^+muscle^*. Rescue of motility phenotypes was weaker in Gly147Arg; *nfu-1^+neurons^*. The wave initiation rate was not changed in Gly147Arg; *nfu-1^+neurons^* compared to Gly147Arg alone, indicating that re-expression of WT *nfu-1* in neurons is insufficient to rescue the increased motility of Gly147Arg animals. Surprisingly, the swimming speed was significantly elevated in both Gly147Arg; *nfu-1^+muscle^* and Gly147Arg; *nfu-1^+neurons^* compared to Gly147Arg animals alone. We do not have a satisfactory explanation for this finding at this time.

Data from the tissue-specific knockouts suggested that *nfu-1^+muscle^* would likely most effectively rescue Gly166Cys motility phenotypes. Re-expression of WT *nfu-1* in either the muscles or neurons of Gly166Cys animals significantly improved all aspects of motility (Fig. 5B-G, Table 1). However, the effects tended to be greater in Gly166Cys; *nfu-1^+muscle^* than in Gly166Cys; *nfu-1^+neurons^* animals, as was most obvious in the wave initiation rate, swimming speed and activity index (Fig. 5B-D, Table 1). The exception to this trend is curling, for which the rescue was more significant in Gly166Cys; *nfu-1^+neurons^* than in Gly166Cys; *nfu-1^+muscle^* (Fig. 5F, Table 1). Together, these data confirm that the necessity for *nfu-1* differs between muscles and neurons and that muscles may be more sensitive than neurons to NFU-1 dysfunction.

### Knockdown of ACh secretion rescues motility phenotypes of the Gly147Arg variant

**Fig. 6.**
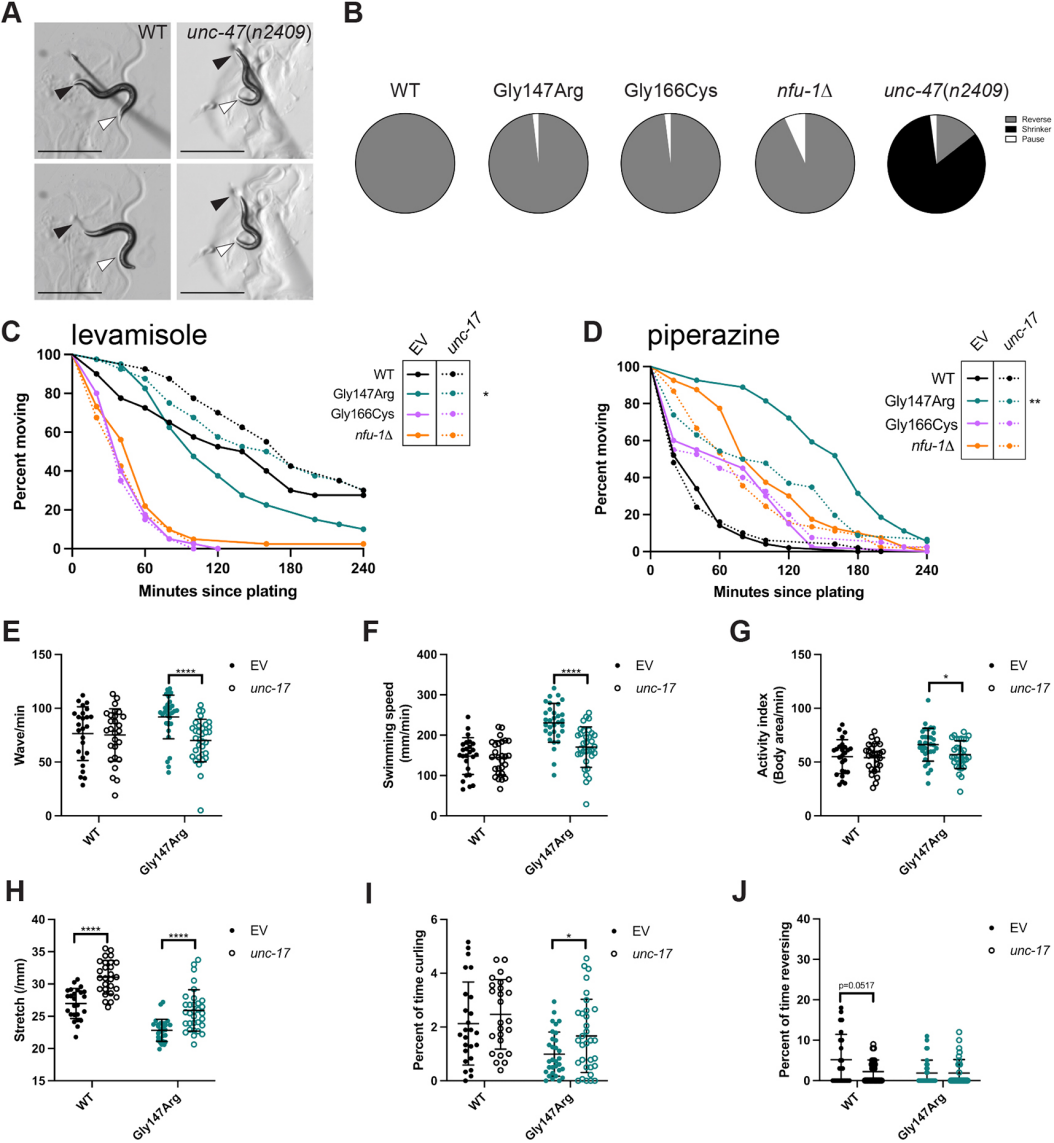
**Knockdown of ACh signaling rescues Gly147Arg motility phenotypes.** (A) Representative images of shrinker analysis. Black arrowheads indicate head position; white arrowheads indicate tail position at time of nose touch (top row). Bottom images represent 1[Supplementary-material sup1]s after nose touch. Arrowheads are in the same position in both images. Shrinker animals [*unc-47(n2409)* as positive control] contract from the anterior body without any posterior movement. WT animals reverse. Scale bars: 500 μm. (B) Shrinker phenotype as represented as percentage of total population (*n*=101-138). (C) Levamisole paralysis curve. Empty vector (EV)-treated samples in solid lines and *unc-17* RNAi interference (RNAi)-treated samples in dotted lines. (*n*=40-41). (D) Piperazine paralysis curve. Solid lines, EV-treated samples; dotted lines, *unc-17* RNAi-treated samples (*n*=40-54). Data plotted as Kaplan–Meier survival curves. (E-J) Swimming phenotypes of EV- or *unc-17* RNAi-treated samples in filled or open circles, respectively (*n*=25-33). (E) Wave rate. (F) Swimming speed. (G) Activity index. (H) Stretch. (I) Percentage of time curling. (J) Percentage of time reversing. Each data point represents an individual animal (*n*=25-33). *P≤0.05; **P≤0.01; ****P≤0.0001 by parallel two-tailed Student's *t*-tests.

Our testing of mutants that specifically impair either cholinergic or GABAergic signaling (Fig. S2, Table S2) indicated that either signaling pathway could cause the paralysis phenotypes observed in the *nfu-1* variants. We thus chose to investigate these pathways more specifically. With dysfunctional GABA signaling, a gentle nose touch characteristically results in the ‘shrinker’ phenotype due to impaired relaxation of body wall muscle (Fig. 6A). In shrinkers, gentle nose touch results in contraction of anterior body muscles but does not stimulate backward movement. Thus, the animal shrinks, unlike WT, which simply reverses. The *nfu-1* variants were tested for the shrinker phenotype, but, unlike the control *unc-47(n2409)*, none showed any difference from WT (Fig. 6B). The *nfu-*1 variants did appear to pause, neither shrinking nor reversing, somewhat more frequently when touched, but this phenotype is likely to be due to altered sensation of physical touch (Kaplan and Horvitz, 1993; Perkins et al., 1986; Ware et al., 1975). Alterations in mechanosensation are the subject of ongoing work and beyond the scope of this study. However, the lack of a shrinker phenotype in the *nfu-1* variants indicated that altered GABA signaling was not the cause of motility phenotypes. The piperazine resistance of Gly147Arg and Gly166Cys was likely to be caused by excessive ACh signaling, such that relaxation was impaired even in the presence of a GABAR agonist.

The paralysis phenotypes of the *nfu-1* variants, and Gly147Arg in particular, are consistent with hypersensitivity to ACh. UNC-17 is the vesicular ACh transporter (Alfonso et al., 1993), and *unc-17* knockdown should decrease ACh secretion due to reduced ACh in secretory vesicles. We first tested this approach with *tom-1(ok285)*, which hypersecretes ACh (Gracheva et al., 2006). Because *C. elegans* neurons are relatively insensitive to RNA interference (RNAi), a procedure to concentrate the RNAi feeding bacteria was employed to enhance knockdown of *unc-17*. This procedure was effective at partially rescuing the *tom-1(ok285)* hypersensitivity to levamisole (Fig. S5, Table S5), validating this approach for knockdown of ACh secretion.

The *nfu-1* variants were treated with the *unc-17* RNAi protocol and then subjected to separate levamisole and piperazine paralysis assays. Knockdown of *unc-17* had no effect on Gly166Cys or *nfu-1*Δ paralysis when the knockdowns were compared to the empty vector control (Fig. 6C,D), indicating that hypersecretion of ACh is not the cause of motor dysfunction in these variants. Surprisingly, RNAi treatment by itself caused a slight rightward shift in the piperazine paralysis curve of *nfu-1*Δ (Fig. 6D compared to Fig. 3D), which we hypothesize is due to the drugs used for RNAi [isopropyl-β-d-thiogalactopyranoside (IPTG) and carbenicillin] or an effect caused by feeding with HT115 bacteria rather than the standard OP50. However, *unc-17* knockdown in Gly147Arg restored sensitivity to levamisole and resistance to piperazine to near-WT levels (Fig. 6C,D; Table S6), indicating that hypersecretion of ACh contributes to motor dysfunction in the Gly147Arg variant. The ability of *unc-17* RNAi to rescue the swimming phenotypes of Gly147Arg was thus tested, and, as expected, nearly all metrics of hyperactivity were restored to WT levels (Fig. 6E-J). These results thus suggested that Gly147Arg enhanced release of ACh, which can be rescued by reducing ACh secretion.

### N-acetyl-L-cysteine cannot rescue neuromuscular signaling defects

We previously showed that treatment with N-acetyl-L-cysteine (NAC), an antioxidant, could extend the lifespan of Gly147Arg, indicating that oxidative stress contributed to the stress phenotypes of this mutant. Oxidative stress is known to affect neuromuscular signaling (Jia and Sieburth, 2021; Kim and Sieburth, 2018, 2019), so the possibility that oxidative stress contributed to the neuromuscular phenotypes of the *nfu-1* variants was explored. Animals were raised on plates containing NAC or H_2_0 (vehicle) until the L4 larval stage and then subjected to levamisole and piperazine paralysis assays. Treatment with NAC had no effect on *nfu-1* variant sensitivity to levamisole or resistance to piperazine (Fig. S6A,B, Table S7). Surprisingly, WT animals treated with NAC were hypersensitive to levamisole without a statistically significant change in sensitivity to piperazine (Fig. S6A,B, Table S7). At the time of writing, we are unaware of any other reports of this effect in WT *C. elegans*, and the mechanism remains unknown. In all, our results demonstrated the tissue-specific effects of *nfu-1* variants on ACh signaling.

## DISCUSSION

**Fig. 7.**
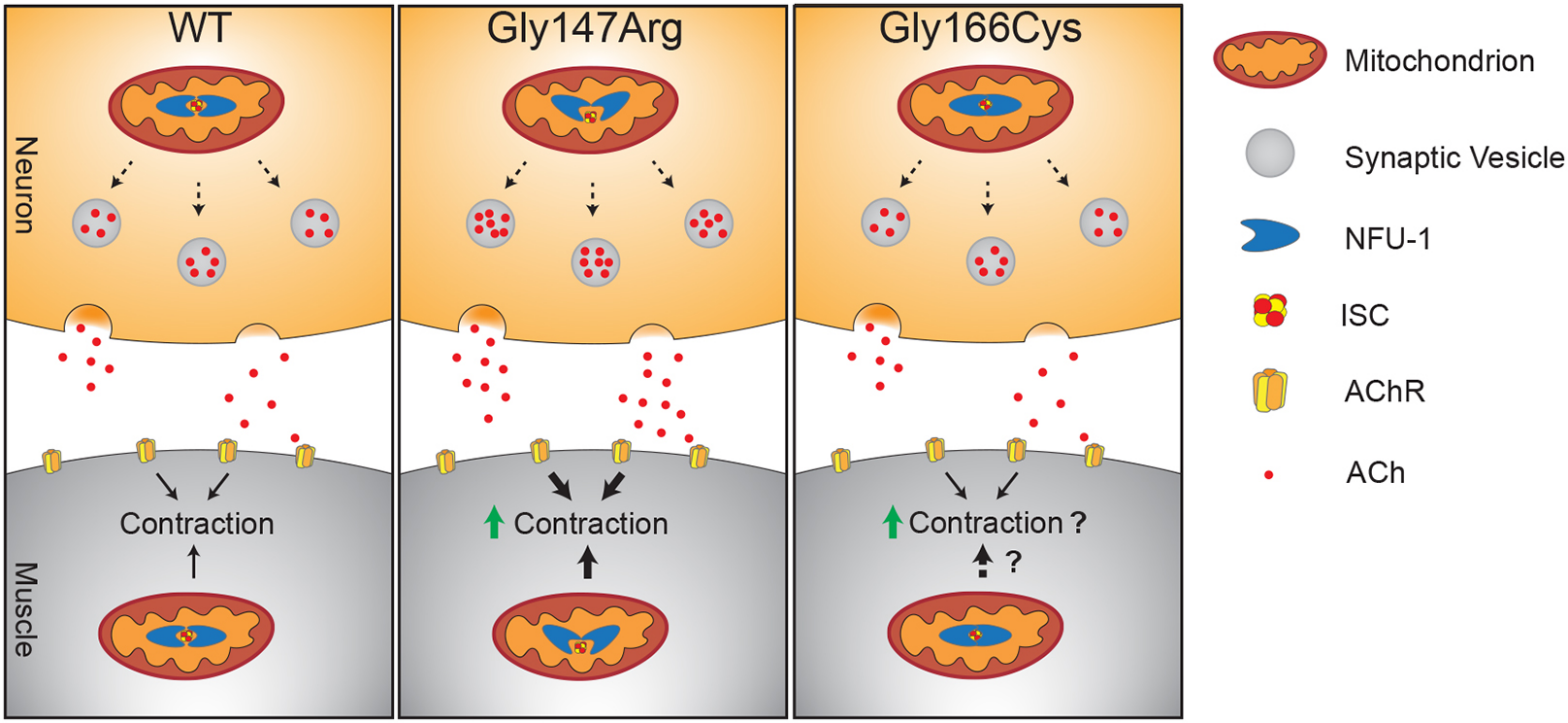
**Summary of findings.** In WT animals, NFU-1 functions normally within neuronal mitochondria, and there are normal levels of ACh packaged into vesicles and secreted in the synapse. Normal mitochondrial function is indirectly necessary for vesicular packaging, indicated by dashed line arrows in neurons. ACh binding to AChRs thereby stimulates contraction. Normal NFU-1 function in muscle mitochondria allows for contraction to occur normally. In Gly147Arg animals, NFU-1 dimerizes around iron–sulfur clusters (ISCs) less efficiently, thereby altering mitochondrial function. By an unknown mechanism, this mitochondrial dysfunction in neurons causes hypersecretion of ACh either through increased packaging of ACh into synaptic vesicles, increased vesicular release or both. Increased ACh signaling increases contractions irrespective of muscular mitochondrial dysfunction. In Gly166Cys animals, NFU-1 fails to release ISCs, thus inhibiting mitochondrial function. Neuronal ACh secretion is unaffected, but muscular contraction is altered by an unclear mechanism (dashed line arrow in muscle). Animals' hypersensitivity to aldicarb and levamisole suggest that contraction is potentiated or increased, consequently causing reduced motility. The nature of contractile defect and mechanism are unknown, indicated by dashed line arrow in muscle and question marks.

Our prior results (Kropp et al., 2021a) and those presented here indicate that patient-specific variants of *nfu-1*, both of which are reduction- or loss-of-function alleles, result in neuromuscular dysfunction, with each *nfu-1* variant resulting in distinct neuromuscular defects (Fig. 7). Both Gly147Arg and Gly166Cys are hypersensitive to the ACh agonists aldicarb and levamisole and resistant to the GABA agonist piperazine. However, they have opposite motility phenotypes: Gly147Arg is hyperactive, whereas Gly166Cys, like *nfu-1*Δ, is hypoactive. Therefore, Gly147Arg could be narrowly defined as having a gain-of-function locomotion phenotype. The mechanistic differences between these variants are very likely due to the specific differences in how these variants coordinated ISCs. We and others have previously shown that the Gly147Arg variant of NFU-1 fails to dimerize as effectively around ISCs, thereby impairing delivery to target proteins and causing excessive oxidative stress (Kropp et al., 2021a; Wesley et al., 2017 a; Wesley et al., 2017b). Gly166Cys, owing to the additional cysteine residue, fails to release ISCs, thereby also impairing delivery to target proteins (Kropp et al., 2021a; Wachnowsky et al., 2017). Thus, although ISC delivery is disrupted in both *nfu-1* alleles, the biological defects differ depending on the specific variant. One explanation could depend on different downstream effects on metabolic function. We previously demonstrated that Gly147Arg shows reduced function of the lipoic acid synthase (human, LIAS; *C. elegans*, LIAS-1), an essential metabolic enzyme and target of NFU1/NFU-1, but to a level sufficient to maintain normal respiratory capacity (Kropp et al., 2021a). In contrast, LIAS-1 function is largely eliminated in Gly166Cys and *nfu-1*Δ, resulting in severely reduced respiratory capacity. This severe loss of respiratory capacity likely impacts muscle cells in a way that the partial reduction of Gly147Arg does not.

### ACh hypersensitivity

Our results showing that RNAi knockdown of the ACh vesicular transporter, UNC-17, suppresses the hypersensitivity of Gly147Arg to levamisole suggest that Gly147Arg enhances the activity of cholinergic signaling, potentially by elevated ACh secretion. As such, Gly147Arg, which is hypersensitive to aldicarb, corresponds to a ‘Hic’ (hypersensitivity to inhibitors of cholinesterase) mutant in line with mutants causing hypersecretion of ACh (Lackner et al., 1999; Miller et al., 1999; Miller et al., 2000; Nurrish et al., 1999; Reynolds et al., 2005; Schade et al., 2005). This interpretation is consistent with ubiquitous *nfu-1* expression in the nervous system, including the ventral nerve cord (Taylor et al., 2021). Other Hic genes are also expressed in the ventral nerve cord, where they act through regulation of diacyl glycerol to stimulate ACh secretion (Lackner et al., 1999; Miller et al., 1999; Miller et al., 2000; Reynolds et al., 2005). That most motility phenotypes were not rescued in Gly147Arg; *nfu-1^+neurons^* complicates our understanding of the role of Gly147Arg in motor neurons despite the strong similarity to Hic phenotypes. It is possible that expression of WT *nfu-1* was too low to overcome the effects of Gly147Arg. As noted above, the molecular mechanism causing potential ACh hypersecretion from neurons in Gly147Arg remains to be discovered, but it likely differs from that of either Gly166Cys or *nfu-1*Δ, which appears to alter the cholinergic response of body wall muscle.

Notably, a recent RNAi screen determined that knockdown of genes involved in ATP synthesis pathways result in hypersensitivity to levamisole (Chaya et al., 2021). These results indicate that disruption of mitochondrial metabolism in body wall muscle is sufficient to induce levamisole hypersensitivity. Our finding that re-expression of WT *nfu-1* in the muscles of both Gly147Arg and Gly166Cys animals rescued many motility phenotypes supports this claim. Furthermore, it is likely that the levamisole hypersensitivity phenotypes of Gly166Cys and *nfu-1*Δ are caused by disrupted mitochondrial metabolism in body wall muscle cells given the nearly complete rescue of motility in the Gly166Cys; *nfu-1^+muscle^* animals.

Another important consideration for these results is the possibility of neurodegeneration contributing to the observed neuromuscular defects. It is well documented in both humans and *C. elegans* that mitochondrial dysfunction including MMDS1 can result in neurodegeneration. Although the integrity of motor neurons was not investigated in this study, such analyses are well established in *C. elegans* (Caldwell et al., 2020; Kropp et al., 2021b) and remain a subject of interest for future work. An intriguing possibility as a source of neurodegeneration in the *nfu-1* variants is ferroptosis, a unique cause of cell death caused by iron toxicity. Our previous work demonstrated that cellular iron is elevated in *nfu-1* variants (Kropp et al., 2021a). Ferroptosis was ruled out as a cause of cell death in the germline, but its potential impact on neurons remains to be evaluated.

### Atypical movement and behavior

All *nfu-1* variants displayed atypical movement. Gly147Arg animals demonstrated hyperactivity on solid medium and in liquid, as indicated by increased rate and speed of crawling or swimming, respectively. As demonstrated by phenotypic rescue by RNAi knockdown of the vesicular ACh transporter, *unc-17*, movement defects of Gly147Arg mutants are likely to be at least partially attributable to hypersecretion of ACh. Exceptions are stretch and time spent curling or reversing, which are not suppressed by *unc-17* RNAi knockdown. Interestingly, neither stretch nor curling was different from WT in basal conditions, yet *unc-17* RNAi significantly elevated both stretch and curling in Gly147Arg compared to empty vector RNAi. In fact, *unc-17* RNAi seems to cause increased stretch regardless of genotype. However, curling while swimming is not a well-characterized phenotype, and thus the exact meaning of the increase with *unc-17* RNAi is unclear. As reversals are generally considered an aspect of foraging behavior (see below), it is possible that the reduced reversals while swimming observed with Gly147Arg are not a primary phenotype of neuromuscular dysfunction and thus cannot be rescued by reducing ACh signaling. Therefore, these data demonstrate that many, but not all, of the Gly147Arg motility phenotypes are caused by ACh hypersecretion.

On solid medium and in liquid, Gly166Cys and *nfu-1*Δ were hypoactive, with multiple atypical patterns of movement. Both variants demonstrated dramatic decreases in wavelength with commensurate increases in amplitude on solid medium, indicating that each body wave was deeper and shorter than those of WT. In liquid, this phenotype appears to have manifested as increased stretch, meaning that each wave was deeper, bringing the head and tail closer together. The cause of this stretch phenotype remains unclear, but could be due to an alteration in the coordinated contraction and relaxation necessary for normal sinusoidal waves. Failure of these variants to relax fully due to a state of constant contraction could explain the hypersensitivity to levamisole, deeper bends and increase in curling observed here.

An unexpected finding was the dramatic increase in reversals of Gly166Cys and *nfu-1*Δ animals when crawling on a solid medium. Reversing is a normal aspect of foraging behavior (also stated above), especially during local searching for food (Calhoun et al., 2014; Gray et al., 2005; Pierce-Shimomura et al., 1999). Reversal behavior is generally considered to be a function of behavioral response to physical or chemical stimuli, which must be integrated to determine whether animals proceed forward, turn, or reverse and change direction (Gray et al., 2005; Pierce-Shimomura et al., 1999; Zariwala et al., 2003; Zhao et al., 2003). When removed from food, *C. elegans* will explore for food in part by chemotaxing toward attractive stimuli including olfactory signals (de Bono and Bargmann, 1998; Fujiwara et al., 2002; Hills, 2004; Sawin et al., 2000). When attractive stimulation is increasing, the rate of reversals is reduced. Thus, the increased incidence of reversals as well as duration of reversals in the *nfu-1* variants, and Gly166Cys and *nfu-1*Δ in particular, suggest a chemosensory defect that causes this behavior. This phenotype is the subject of ongoing investigation.

### NAC effects on neuromuscular signaling

Given the ability of antioxidants to extend the lifespan of Gly147Arg (Kropp et al., 2021a), we performed experiments to determine whether oxidative stress contributed to Gly147Arg motility phenotypes. Work in other systems has demonstrated that oxidative stress can negatively affect the function of AChRs, especially in neurodegenerative diseases such as Alzheimer's disease (Cacanyiova et al., 2013; Campanucci et al., 2008; Guan, 2008; Krishnaswamy and Cooper, 2012). Conversely, the stress (including oxidative) regulator SKN-1 can negatively regulate ACh release (Staab et al., 2013; Staab et al., 2014). This mechanism likely does not contribute to the cholinergic hypersensitivity of *nfu-1* mutants given that *nfu-1* RNAi activates SKN-1 (Kropp et al., 2021a), which would be expected to reduce ACh secretion. The most surprising finding was that the antioxidant, NAC, significantly increased the levamisole sensitivity of WT animals (Fig. 6A). At the time of writing, we are unaware of any previous reports of this finding. However, there is evidence that NAC can increase the contractility of skeletal muscle in other systems (Okay et al., 2010), potentially through inhibition of AChE (Abdel-Wahab and Moussa, 2019; Costa et al., 2015). Such inhibition would potentiate cholinergic signals, thereby increasing sensitivity to levamisole. Perhaps WT animals are sensitive to this level of regulation whereas the hypersensitivity of the *nfu-1* variants masks any effect of NAC.

### Relevance to human MMDS1

As noted above, dystonia is a shared phenotype of nearly all MMDS1 patients, so this work is applicable to human disease biology. The most significant finding is that the motility phenotypes of Gly147Arg can be rescued by reducing ACh signaling. Importantly, one MMDS1 patient is homozygous for Gly189Arg, the NFU1 orthologous residue of Gly147Arg modeled here. This individual presented with hypertonia rather than hypotonia, making the results here directly translatable from *C. elegans* to humans (Uzunhan et al., 2020). Furthermore, the hypertonia of this patient strengthens the conclusion that Gly147Arg/Gly189Arg, despite having reduced ISC delivery function, could result in elevated ACh secretion from motor neurons. This individual is also one of the few MMDS1 patients alive at the time of the case report, further indicating that Gly147Arg/Gly189Arg is a comparatively less severe variant than other documented NFU-1/NFU1 variants. These results suggest that a cholinergic antagonist could be relevant as a therapeutic intervention for MMDS1 patients with the Gly189Arg variant.

In contrast to the Gly147Arg variant, the phenotypic effects of the Gly166Cys variant are more consistent with a full loss-of-function of NFU-1 activity. This variant is not only the most common allele documented in MMDS1, but it is also more similar to other common NFU1 variants than Gly147Arg (Kropp et al., 2021a). These data, combined with data from *nfu-1*Δ and Gly166Cys, strongly suggest that other complete loss-of-function variants have reduced capacity to respond to cholinergic signaling, thereby causing hypotonia. As noted above, we chose to analyze these variants in isolation as homozygotes to best investigate the unique characteristics of each variant. However, approximately half of MMDS1 patients are compound heterozygous for multiple variants. As demonstrated in one recent study of endolysosomal trafficking dysfunction (van der Welle et al., 2021), analysis of compound heterozygous variants is highly effective in *C. elegans*. Such an approach would be possible with *nfu-1* variants to better elucidate the genotype/phenotype relationship of individual MMDS1 patients. Although more research is needed to understand the molecular mechanisms of all NFU1/NFU-1 variants causing neuromuscular dysfunction, this work has identified cholinergic signaling as a prime target and developed tools for its investigation.

## MATERIALS AND METHODS

### Animal maintenance

Animals were maintained following standard procedures (Brenner, 1974) on MYOB plates seeded with OP50 bacteria. All *nfu-1* variants were maintained as heterozygotes balanced with *tmC5*. Any modifications to this process as required for experiments are described below. All strains used are listed in Table S8.

### CRISPR gene editing

CRISPR gene editing was carried out as in Kropp et al. (2021a). In brief, CRISPR injection mixes were prepared and carried out by injection into the syncytial gonad with *dpy-10* as a co-injection marker as described in Paix et al. (2017). Guide RNAs were purchased from Horizon Discovery, and repair sequences were either purchased from IDT or generated by PCR using Phusion polymerase (NEB). FRT sites around the endogenous *nfu-1* locus were added sequentially with the 5′ sites added first. Owing to linkage of the *nfu-1* locus with the *bqSi495* and *bqSi506* insertion site on chromosome IV, the FRT sites were inserted into each strain independently, resulting in the identical *nfu-1(av246)* allele in each genetic background. For transgenic rescue constructs, the SKI LODGE system of single-copy insertions was utilized (Silva-García et al., 2019). All *C. elegans nfu-1* constructs utilized the entire *nfu-1* genomic locus (exons and introns), whereas only the cDNA was inserted for the human *NFU1* rescue construct. Injection mixes were prepared as described in Silva-García et al. (2019), with repair constructs at a concentration of ∼500 ng/μl in the injection mix. The *nfu-1* or *NFU1* sequences were first inserted into the appropriate tissue-specific driver strain (WBM1126, WBM1140, WBM1456). All CRISPR constructs were sequenced for accuracy, and at least two lines were independently generated for each allele. Alleles containing the tissue-specific *nfu-1* or *NFU1* rescue constructs were subsequently crossed into the appropriate *nfu-1* variant, and transgenes were homozygosed prior to analysis. Guide RNA, repair sequences and primers for generation of repair sequences are listed in Table S9.

### Video capture

Videos were captured on a Nikon SMZ1245 microscope equipped with a Hamamatsu Orca Flash 4.0 LT camera controlled by Nikon Elements BR software. Videos were 1[Supplementary-material sup1] min in length and captured at 33 frames/s for swimming or 7.5 frames/s for crawling. Videos were written and saved as MP4 files.

For swimming analysis, sample preparation included picking single animals from a culture plate into a watch glass containing M9 buffer to remove excess bacteria. Animals were then picked from the watch glass to a droplet of M9 buffer on a microscope slide and imaged immediately as described above. One or two animals were imaged per individual video.

For crawling analysis, around five animals were picked directly from a culture plate to an agarose pad. Excess bacteria were removed with a platinum pick.

### Confocal microscopy

Confocal microscopy was carried out using a Nikon Eclipse Ti2 microscope equipped with a Yokagawa CSUX-1 spinning disk and Photometrics Prime95B camera using Nikon NiS Elements acquisition software. Images were captured in widefield at 10× magnification.

Induction of fluorescent reporters was achieved by a 30-min heat shock of L4 samples at 34°C followed by 4 h of recovery at 20°C. Live imaging was carried out by mounting samples on 2% agarose pads and anesthetized with 5 mM levamisole. Samples were imaged through a #1 cover glass sealed with nail polish.

### Motility analysis

Movement phenotypes were assessed with WormLab software purchased from MBF Biosystems (Restif et al., 2014). MP4 files were imported and adjusted for accurate identification of animals. Scale was either 4.81 or 2.18 µm/pixel, and threshold was either 215 or 195 depending on zoom used. Background smoothing was set to 10 for all samples. Width fitting and backtracking were enabled. Following tracking, each track was manually screened for accuracy and repaired (switching head or tail) if the head of the animal was misidentified.

### Shrinker analysis

Animals for shrinker analysis were gently touched on the nose with an eyelash. Responses were scored as either a reversal (whole body reversal), shrinker (contraction of body without movement) or pause (stop in motion without shrinking or reversal).

### RNAi

*C. elegans* were treated with RNAi from the updated Ahringer library (Fraser et al., 2000) expressed in HT115(DE3) bacteria. To increase RNAi expression in neurons, a protocol modified from Timmons et al. (2001) was used to concentrate bacteria. RNAi cultures were concentrated as follows: 3 ml LB [100 mg/ml ampicillin (amp)] medium inoculated with RNAi or empty vector bacterial clone. Cultures were grown for 16 h at 37°C in a drum roller. Then, 100 µl of this culture was inoculated into 5 ml LB (100 mg/ml amp) and incubated for 4 h at 37°C in a drum roller. Following this incubation, IPTG was added to a final concentration of 1 mM, and cultures were incubated for another 4 h at 37°C in a drum roller. Following this incubation, cultures were centrifuged for 10 min at 3750 ***g*** in a benchtop centrifuge. The supernatants were aspirated, and bacterial pellets were resuspended in 2 ml LB (100 mg/ml amp) medium. This culture was seeded onto MYOB plates containing 4 mM IPTG and 100 mg/ml amp. Bacteria were allowed to dry for 48 h prior to use. All plates and cultures were prepared fresh for each experiment.

L4 hermaphrodites were plated onto RNAi plates prepared as above. After 24 h, these adult animals (P_0_) were plated onto a fresh RNAi plate for a 2-h egg lay. P_0_ animals were removed after 2 h, and embryos were allowed to hatch and develop. Animals were used for experiments when they reached the L4 stage, which varied depending on genotype. Data were accumulated from a minimum of three independent experiments.

### NAC treatment

MYOB plates containing 5 mM NAC were prepared fresh for each experiment. NAC was added immediately prior to pouring. Volume-matched vehicle plates (0.8% H_2_O) were simultaneously prepared. Plates were seeded with OP50 bacteria and allowed to dry for 48 h prior to use. L1-arrested larvae (see below) were plated onto NAC or vehicle plates and allowed to grow to the L4 stage, at which point they were used in paralysis assays. Data were accumulated from a minimum of three independent experiments.

Arrested L1 larvae were obtained through sodium hypochlorite treatment of gravid adult hermaphrodite animals and subsequent incubation in S-basal buffer overnight.

### Paralysis assays

Paralysis assays were all performed over 4 h with timepoints every 15 or 20 min depending on assay. Animals were plated at time 0, and assessed for paralysis at the indicated timepoints by touching three times (midbody, head, tail) with an eyelash pick. Animals were considered paralyzed when no response occurred from all three touches.

Drug plates were prepared the day before each assay. Drugs were added to liquid MYOB medium immediately prior to pouring. The final concentration of each drug was as follows: aldicarb, 1 mM (stock in 70% EtOH); levamisole, 40µM (stock in in H_2_O); piperazine, 150 mM (stock in 100% EtOH). Data were accumulated from a minimum of three independent experiments.

### Cell sorting

To generate synchronized cultures, embryos were obtained by hypochlorite treatment of adult hermaphrodites and allowed to hatch in M9 buffer overnight (16-23 h). L1-arrested larvae were then grown on 150 mm 8P (peptone-enriched) plates seeded with NA22 bacteria for 39-43 h at 23°C to promote rapid expansion of the population (Spencer et al., 2014; Taylor et al., 2021; Zhang et al., 2011). For the heat-shock treatment, the 8P plates were placed in a single layer in a 34°C incubator for 30 min and then transferred to a 20°C incubator to recover for 3 h. After treatment, populations were L4 larvae and young adults.

Cell dissociation and FACS were conducted as previously described (Spencer et al., 2014; Taylor et al., 2021; Zhang et al., 2011). Briefly, worms were washed from the plates and separated from the bacteria through slow speed spins in cold M9, yielding two ∼300 µl wet pellets of animals. Pellets were transferred to 1.7 ml tubes and treated with 2× volumes of SDS-DTT (20 mM HEPES, 0.25% SDS, 200 mM DTT, 3% sucrose, pH 8.0). Egg buffer was added to stop the reaction. The worms were washed in 1.0-1.5 ml egg buffer, resuspended in 2× volumes of 15 mg/ml pronase and dissociated by pipetting. The pronase digestion was stopped with 1.0-1.5 ml L-15-10 (Gibco L-15 medium, 10% fetal bovine serum, 0.5% penicillin/streptomycin). After centrifugation, cells were resuspended in 1 ml cold egg buffer and separated from large debris with a low-speed spin (100 rcf for 2.5 min at 4°C). The supernatant was passed through a 35-μm filter into a collection tube. The pellet from the low-speed spin was resuspended and re-pelleted at low speed to recover additional cells in the supernatant, which were passed through the 35-µm filter and added to the collection tube.

FACS was performed on the cell suspension in the collection tube on a BD FACSAria™ III equipped with a 70-μm-diameter nozzle. Then, 4′,6-diamidino-2-phenylindole (DAPI; 1 mg/ml) was added to the sample (final concentration of 1 µg/ml) to label dead and dying cells. Cells were separated for either GFP or mCherry expression. Sorted cells were collected directly into Trizol LS and stored at −80°C for RNA extractions.

### Gene expression analysis

RNA extraction, cDNA synthesis and qRT-PCR were carried out as in Kropp et al. (2021a). In brief, RNA was extracted in Trizol Reagent (Thermo Fisher Scientific) and purified with a Zymo QuickRNA MiniPrep kit (Zymo). Synthesis of cDNA was carried out with iScript cDNA Synthesis reagents (Bio-Rad). qRT-PCR was carried out with SYBR Select Master Mix (Thermo Fisher Scientific) on a Bio-Rad CFX96 thermocycler. Primers used in this study are listed in Table S10.

### Statistical analyses

Data plotting and statistical analyses were carried out with GraphPad Prism software (version 9.1.2). Sample sizes and statistical methods used are indicated in figure and table legends. Unless otherwise stated, individual data points are plotted as mean±s.d. Paralysis assays are plotted as Kaplan–Meier survival curves with data aggregated from at least three independent experiments.

## Supplementary Material

10.1242/dmm.049594_sup1 Supplementary information
