# Supplementary material for: Patient-specific variants of NFU1/NFU-1 disrupt cholinergic signaling in a model of multiple mitochondrial dysfunctions syndrome 1
Source: Dis Model Mech. 2023 Feb 1;16(2):dmm049594. doi: 10.1242/dmm.049594 (PMC9922734; doi:10.1242/dmm.049594)
Supplement: Supplementary information [file dmm-16-049594-s1.pdf]

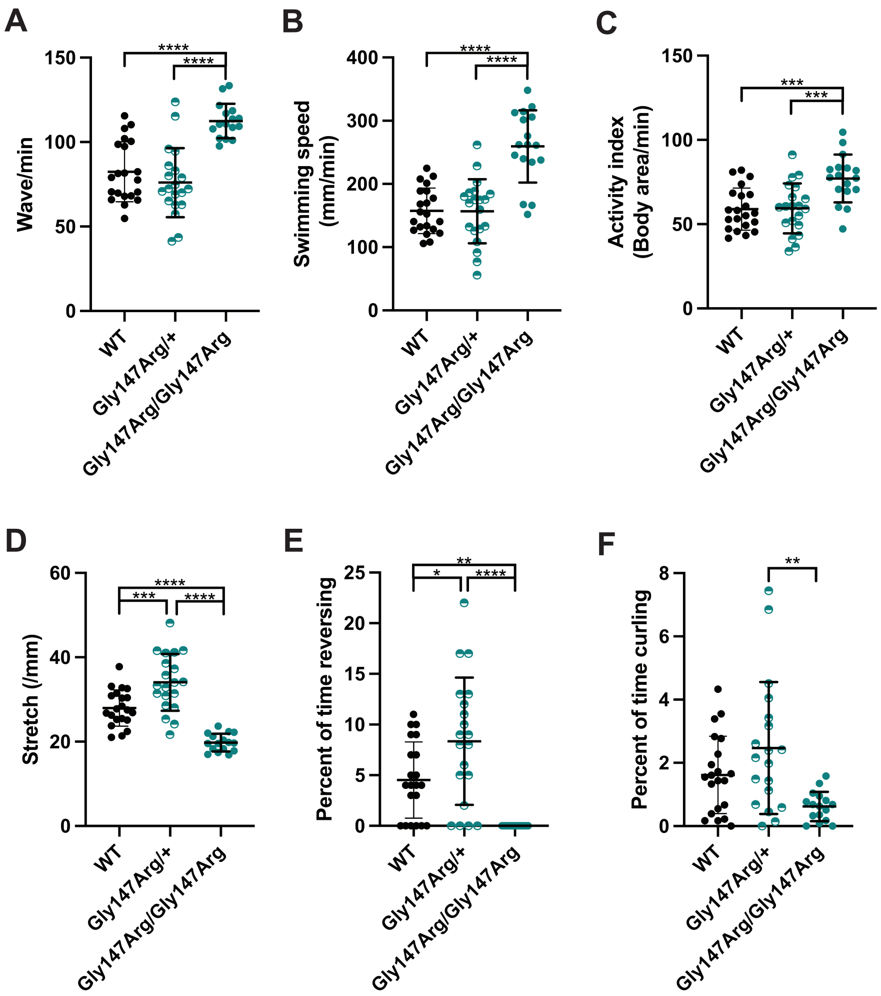

Fig. S1. Analysis of Gly147Arg heterozygote swimming behavior

(A) Wave rate. (B) Swimming speed. (C) Activity Index. (D) Stretch. (E) Percent of time curling. (F) Percent of time reversing of L4 WT, Gly147Arg heterozygotes (Gly147Arg/+) and Gly147Arg homozygotes (Gly147Arg/ Gly147Arg). Each data point represents an individual animal (n=17-21). *: p≤0.05; **: p≤0.01; ***: p≤0.001; ****: p≤0.0001 by one-way ANOVA with Tukey correction for multiple comparisons.

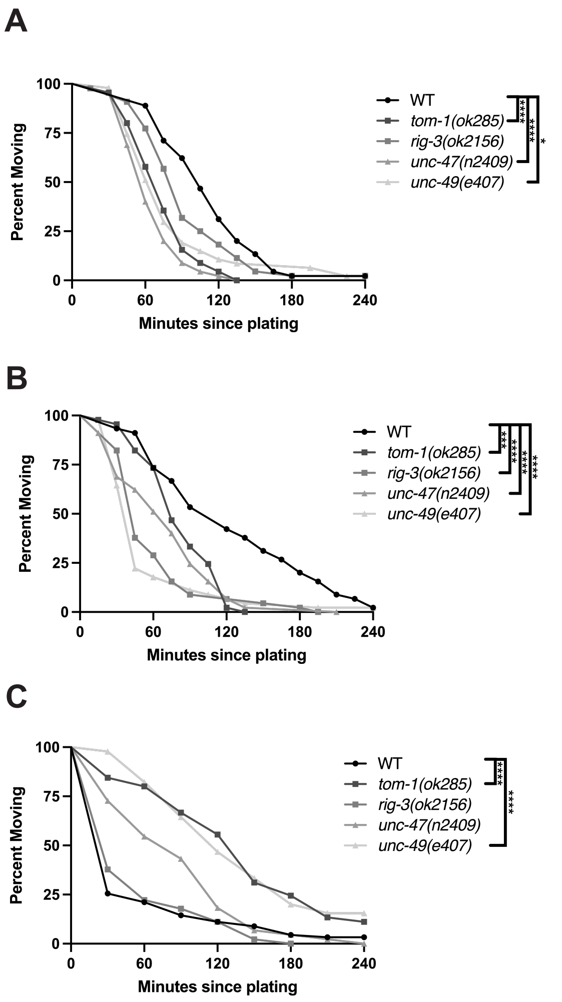

Fig. S2. Paralysis curves of synaptic mutants

(A) Aldicarb paralysis curve (n=44-47). (B) Levamisole paralysis curve (n=45). (C) Piperazine paralysis curve (n=44-90). Data plotted as Kaplan-Meyer survival curves. *: p≤0.05; ***: p<0.001; ****: p≤0.0001 by Log-Rank analysis with Bonferroni correction for multiple comparisons.

Disease Models & Mechanisms • Supplementary information

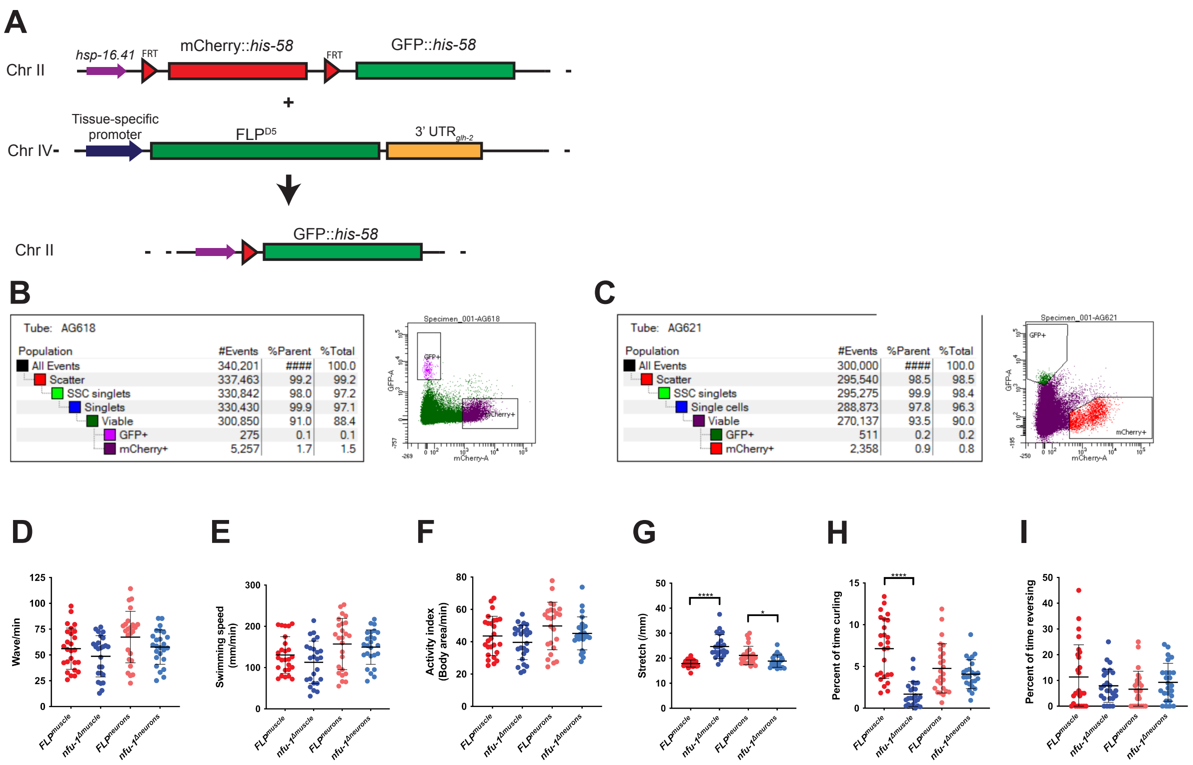

Fig. S3. Cell sorting profiles from tissue-specific *nfu-1* knockouts

(A) Schematic of transgenic reporter of recombination modified from (Muñoz-Jiménez et al., 2017). Transgene is driven by *hsp-16.41* promoter which is induced by heatshock (see methods). Transgene expression produces a nuclear mCherry in cells where FLP is not active. In cells where FLP is active, the mCherry sequence is excised allowing for expression of nuclear GFP. (B,C) BD FACSAria^{TM} III output for sorted *nfu-1*$^{\Delta muscle}$ (B) and *nfu-1*$^{\Delta neurons}$ (C) samples. Only final gating shown. (D) Wave rate. (E) Swimming speed. (F) Activity Index. (G) Stretch. (H) Percent of time curling. (I) Percent of time reversing of L4 animals. Each data point represents an individual animal (n=23-27). For D-I, p≤0.001; ****: p≤0.0001 by parallel Student's t-tests.

Disease Models & Mechanisms • Supplementary information

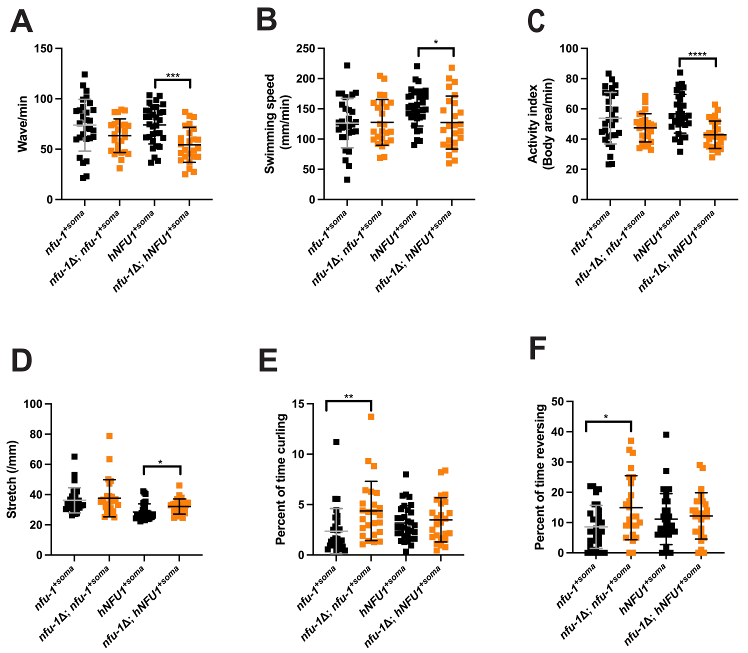

Fig S4. Somatic re-expression of WT *nfu-1* or *hNFU1* alone and in *nfu-1*Δ

(A) Wave rate. (B) Swimming speed. (C) Activity index. (D) Stretch. (E) Percent of time curling. (F) percent of time reversing. Each data point represents an individual animal (n=25-32). For *: p≤0.05; **: p≤0.01; ***: p≤ 0.001; ****: p≤0.0001 from respective transgene-only control by Students' t-test.

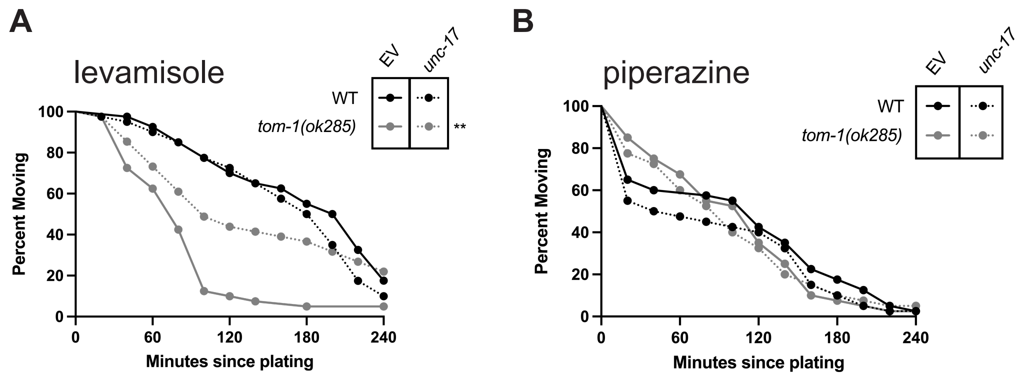

Fig. S5. Rescue of ACh hypersecretion phenotype with *unc-17* RNAi

Levamisole paralysis curve (n=40-41). (B) Piperazine paralysis curve (n=40). EV treated samples in solid lines and *unc-17* RNAi treated samples in dotted lines. Data plotted as Kaplan-Meyer survival curves. **: p≤0.01 by Log-Rank analysis.

Disease Models & Mechanisms • Supplementary information

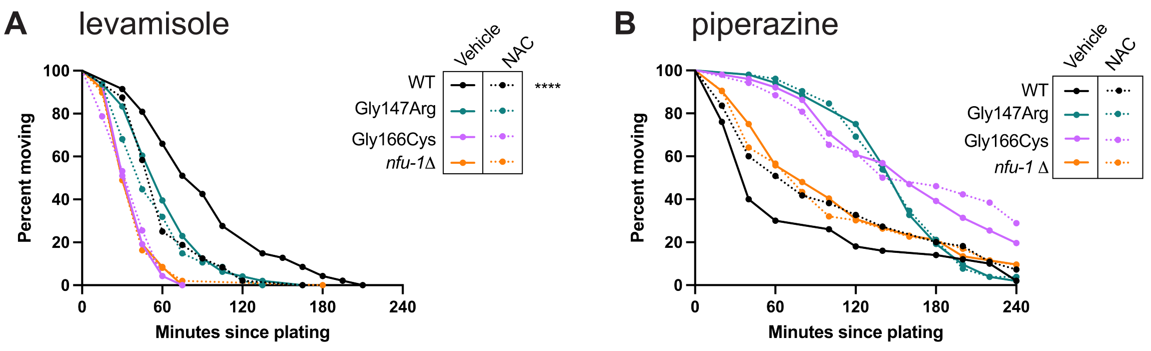

Fig. S6. NAC does not ameliorate altered sensitivities of *nfu-1* variants to levamisole or piperazine

(A) Levamisole paralysis curve (n=47-49). (B) Piperazine paralysis curve (n=50-55). Vehicle (H_2O) in solid lines and NAC in dotted lines. Data plotted as Kaplan-Meyer survival curves. ****: p≤0.0001 by Log-Rank analysis with Bonferroni correction for multiple comparisons.

Disease Models & Mechanisms • Supplementary information

Table S1. Details of paralysis assays in Fig. 3

Significance from WT determined for each variant by Log-Rank analysis with Bonferroni correction for multiple comparisons.

Strain/variant	Aldicarb Sample size	Aldicarb Median time to paralysis	Aldicarb Adjusted p-value	Levamisole Sample size	Levamisole Median time to paralysis	Levamisole Adjusted p-value	Piperazine Sample size	Piperazine Median time to paralysis	Piperazine Adjusted p-value
WT	45	105	NA	65	120	NA	90	30	NA
Gly147Arg	45	90	0.0138	63	60	0.04682	89	150	<0.0001
Gly166Cys	43	90	0.0009	59	30	<0.0001	94	150	<0.0001
nfu-1 Δ	44	75	0.0405	66	30	<0.0001	89	30	0.3309

Table S2. Details of paralysis assays in Fig. S1

Significance from WT determined for each variant by Log-Rank analysis with Bonferroni correction for multiple comparisons. Protein function, physiological effect of mutant, and expected curve shift are indicated for each mutant. NA: not applicable; NE: no effect

Strain/mutant	Protein function	Net effect	Aldicarb Expected curve shift	Aldicarb Sample size	Aldicarb Median time to paralysis	Aldicarb Adjusted p-value of curve difference from WT	Levamisole Expected curve shift	Levamisole Sample size	Levamisole Median time to paralysis	Levamisole Adjusted p-value of curve difference	Piperazine Expected curve shift	Piperazine Sample Size	Piperazine Median time to paralysis	Piperazine Adjusted p-value of curve difference from WT
WT				45	105	NA		45	120	NA		90	30	NA
tom-1(ok285)	Tomosyn	ACh hypersecretion	Left	45	75	<0.0001	Left	45	75	0.0003	NE or Right	45	150	<0.0001
rig-3(ok2156)	Cell surface immunoglobulin	Increased AChR (ACR-16) abundance	Left	45	90	0.2124	Left	45	45	<0.0001	NE or Right	45	30	0.3791
unc-47(n2409)	VGAT	GABA hyposecretion	Left	44	60	<0.0001	Left	45	75	<0.0001	Right	44	90	0.11
unc-49(e407)	$GABA_A$ Receptor	Decreased GABAR abundance	Left	47	75	0.0104	Left	45	45	<0.0001	Right	45	120	<0.0001

Table S3. Gene expression results from sorted *nfu-1*$^{\Delta muscle}$ and *nfu-1*$^{\Delta neurons}$ samples

Relative expression was determined by the Δct method with comparison to *act-1* as the housekeeping gene. Since this is Δ ct, larger values indicate lower expression. ND: not detected; NA: not assessed.

		Δct (from *act-1*) *nfu-1*	*unc-17*	*unc-3*	*cha-1*	*cho-1*
nfu-1 $^{\Delta muscle}$	GFP+	ND	NA	NA	NA	NA
	mCherry +	11.27	NA	NA	NA	NA
nfu-1 $^{\Delta neurons}$	GFP+	ND	4.46	5.93	1.61	1.57
	mCherry +	13.61	10.94	ND	8.97	9.10

Table S4. Details of paralysis assays in Fig. 4

For each tissue-specific *nfu-1* knockout, difference was calculated from the appropriate control strain (*FLP*^{muscle} for *nfu-1*$^{\Delta muscle}$; *FLP*^{neurons} for *nfu-1*$^{\Delta neurons}$) by Log-Rank analysis.

Strain	Levamisole Sample size	Levamisole Median time to paralysis	Levamisole p-value from Control	Piperazine Sample size	Piperazine Median time to paralysis	Piperazine p-value from Control
FLP ^{muscle}	45	105	0.0001	42	30	0.2041
nfu-1 $^{\Delta muscle}$	45	60		43	15	
FLP ^{neurons}	45	75	0.7062	43	22.5	0.1829
nfu-1 $^{\Delta neurons}$	45	75		44	22.5	

Table S5. Details of paralysis assays in Fig. S3

For each strain, difference was determined between EV and *unc-17* RNAi treatment by Log-Rank analysis. EV: empty vector; RNAi: RNA interference.

Strain/mutant	Levamisole RNAi Treatment EV Sample size	Levamisole EV Median time to paralysis	Levamisole *unc-17* Sample size	Levamisole *unc-17* Median time to paralysis	Levamisole p-value from EV	Piperazine RNAi Treatment EV Sample size	Piperazine EV Median time to paralysis	Piperazine *unc-17* Sample size	Piperazine *unc-17* Median time to paralysis	Piperazine p-value from EV
WT	40	210	40	190	0.2138	40	120	40	50	0.3928
tom-1(ok285)	40	80	41	100	0.0027	40	120	40	100	0.8663

Table S6. Details of paralysis assays in Fig. 5

For each strain, difference was determined between EV and *unc-17* RNAi treatment by Log-Rank analysis. EV: empty vector; RNAi: RNA interference.

Strain/mutant	Levamisole RNAi Treatment EV Sample size	Levamisole EV Median time to paralysis	Levamisole *unc-17* Sample size	Levamisole *unc-17* Median time to paralysis	Levamisole p-value from EV	Piperazine RNAi Treatment EV Sample size	Piperazine EV Median time to paralysis	Piperazine *unc-17* Sample size	Piperazine *unc-17* Median time to paralysis	Piperazine p-value from EV
WT	40	150	40	180	0.2545	50	40	40	20	0.2438
Gly147Arg	40	100	40	170	0.0157	54	180	46	90	0.0026
Gly166Cys	40	40	40	40	0.6579	40	80	40	60	0.7361
nfu-1 Δ	41	60	40	40	0.2939	40	90	45	80	0.1546

Table S7. Details of paralysis assays in Fig. 6

For each strain, difference was determined between vehicle (H_2O) and NAC treatment by Log-Rank analysis. NAC: N-acetyl-L-cysteine.

Strain/variant	Levamisole Vehicle Sample size	Levamisole Vehicle Median time to paralysis	Levamisole NAC Sample size	Levamisole NAC Median time to paralysis	Levamisole p-value from Vehicle	Piperazine Vehicle Sample size	Piperazine Vehicle Median time to paralysis	Piperazine NAC Sample size	Piperazine NAC Median time to paralysis	Piperazine p-value from vehicle
WT	47	90	48	60	<0.0001	50	40	55	80	0.1126
Gly147Arg	48	60	47	45	0.2534	52	160	52	160	0.9934
Gly166Cys	47	45	47	45	0.9725	51	160	52	160	0.4273
nfu-1 Δ	47	30	49	30	0.9715	52	80	53	80	0.7867

Disease Models & Mechanisms • Supplementary information

Table S8. Strains used in this study

Strain name, genotype, and source indicated

Strain	Genotype	Source
N2	WT	CGC
AG377	*nfu-1(av115 [Gly147Arg])/tmC5* IV	Kropp et al., 2021
AG561	*nfu-1(av56 [Gly166Cys])/tmC5* IV	Kropp et al., 2021
AG440	*nfu-1(av167 [Δ])/tmC5* IV	Kropp et al., 2021
RB1712	*rig-3(ok2156)* X	CGC
VC223	*tom-1(ok285)* I	CGC
CB407	*unc-49(e407)* III	CGC
MT6201	*unc-47(n2409)* III	CGC
BN503	*bqSi294 [hsp16.41p::FRT::mCherry::his-58::FRT::GFP::his-58 + unc-119(+)]* II; *bqSi495 [myo-3p::FLP D5 unc-119(+)]* IV.	CGC
BN507	*bqSi294 [hsp16.41p::FRT::mCherry::his-58::FRT::GFP::his-58 + unc-119(+)]* II; *bqSi506 [rgef-1p::FLP D5 + unc-119(+)]* IV	CGC
AG618	*nfu-1(av246[FRT::nfu-1::FRT])* IV; *bqSi294 [hsp16.41p::FRT::mCherry::his-58::FRT::GFP::his-58 + unc-119(+)]* II; *bqSi495 [myo-3p::FLP D5 unc-119(+)]* IV	This study
AG621	*nfu-1(av246[FRT::nfu-1::FRT])* IV; bqSi294 *[hsp16.41p::FRT::mCherry::his-58::FRT::GFP::his-58 + unc-119(+)]* II; *bqSi506 [rgef-1p::FLP D5 + unc-119(+)]* IV	This study
WBM1126	*wbmIs61 [myo-3p::3XFLAG::dpy-10 crRNA::unc-54 3'UTR] I*	CGC
WBM1140	*wbmIs65 [eft-3p::3XFLAG::dpy-10 crRNA::unc-54 3'UTR] V*	CGC
WBM1456	*wbmIs127[rab-3p::3XFLAG::dpy-10 crRNA::rab-3 3'UTR] V*	Gift from Mair lab
AG730	*avIs284 [myo-3p::nfu-1::unc-54 3' UTR] I*	This study
AG731	*avIs285 [rab-3p::nfu-1::rab-3 3' UTR] V*	This study
AG728	*avIs286 [eft-3p::nfu-1::unc-54 3'UTR] V*	This study
AG729	*avIs287 [eft-3p::hNFU1::unc-54 3'UTR] V*	This study
AG736	*nfu-1(av56 [Gly166Cys])/tmC5 IV; avIs284 [myo-3p::nfu-1::unc-54 3'UTR] I*	This study
AG735	*nfu-1(av56 [Gly166Cys])/tmC5 IV; avIs285 [rab-3p::nfu-1::rab-3 3' UTR] V*	This study
AG737	*nfu-1(av115 [Gly147Arg])/tmC5 IV; avIs284 [myo-3p::nfu-1::unc-54 3'UTR] I*	This study
AG723	*nfu-1(av115 [Gly147Arg])/tmC5 IV; avIs285 [rab-3p::nfu-1::rab-3 3' UTR] V*	This study
AG724	*nfu-1(av167 [Δ])/tmC5 IV; avIs286 [eft-3p::nfu-1::unc-54 3'UTR] V*	This study
AG727	*nfu-1(av167[Δ])/tmC5 IV; avIs287 [eft-3p::hNFU1::unc-54 3'UTR] V*	This study

Table S9. CRISPR sequences

All sequences oriented 5’ to 3’

Guide and repair sequences

Gene/target	Guide sequence	Repair oligo
nfu-1 5' FRT site	UUUGUACAAGCAGGUGA<br>UAC	ACAAGACTGAACCGGAGCTTTGTA<br>CAAGCAGGTGAGAAGTTCCTATTC<br>TCTAGAAAGTATAGGAACTTCTAC<br>TGGTTCCAGAAATTCTTTCATTTTT<br>TAAAATT
nfu-1 3' FRT site	CGAAUCACGUCAAAGUU<br>CAA	TGAAAGTCGTATTAGTAGAATTGG<br>TTTATGACTCTGAAATCATCGAATC<br>ACGTCAAAGTTGAAGTTCCTATTC<br>TCTAGAAAGTATAGGAACTTCCAA<br>CGGCCCAGTCCAAATCAGTGATAT<br>CAGTTAAAAGACTTATCACCCGGA<br>AAAGTGGAA
dpy-10	UACCAUAGGCACCACGA<br>G	CACTTGAACTTCAATACGGCAAGA<br>TGAGAATGACTGGAAAC<br>CGTACCGCATGCGGTGCCTATGG<br>TAGCGGAGCTTCACATG<br>GCTTCAGACCAACAGCCTAT

Primers for generation of SKI LODGE repair sequences

Target transgene	Forward/Reverse	Sequence
avIs284	For.	CACCTTTTACCGTCTAATTTTCAG<br>GGCAGGGAGCCATCAAACCCACG<br>ACCACTAGATCCATATGATTTCATC<br>AAAAATGCTCACAAG
	Rev.	AGCATGTAGGGATGTTGAAGAGT<br>AATTGGACTTAGAAGTCAGAGGCA<br>GGGCGCGAGATGTTAGTCTTTGA<br>TTCCTTTTGATTGC
avIs285	For.	TGCTCTTTTAAAATAAATCTACAGT<br>AGCCCTATTTTCAGATGACAAGTT<br>TGTACCCCGGGATGATTTCATCAA<br>AAATGCTCACAAG
	Rev.	GCGTTTGGAATTTGGGAAAATTTT<br>GAGTTTTTATAGATAGTATAATAGA<br>ACGTAGAATTTTTAGTCTTTGATTC<br>CTTTTGATTGC
avIs286	For.	CGCACTCTTCTTACTTTAAATTAAA<br>TTGTTTTTTTTTCQGTTGGGAAAC<br>ACTTTGCTCAATGATTTCATCAAAA<br>ATGCTCACAAG
	Rev.	Same as *avIs284*
avIs287	For.	CGCACTCTTCTTACTTTAAATTAAA<br>TTGTTTTTTTTTCQGTTGGGAAAC<br>ACTTTGCTCAATGATTTCATCAAAA<br>ATGCTCACAAG
	Rev.	AGCATGTAGGGATGTTGAAGAGT<br>AATTGGACTTAGAAGTCAGAGGCA<br>GGGCGCGAGATGTTAAGGTGAGT<br>TTGCTTCTTTTTCATC

Disease Models & Mechanisms • Supplementary information

Table S10. qRT-PCR primers used in this study

Forward and reverse primers indicated. All sequences oriented 5' to 3'

Target gene	Forward/Reverse	Sequence (5' - 3')
act-1	For.	CAGAAGGAAATCACCGCTCTT
	Rev.	ATAGATCCTCCGATCCAGACG
nfu-1	For.	GTCAACAGCTTCTCCCAGACG
	Rev.	GCTTCACTCCGTCGACTCG
unc-17	For.	CACTGATTCTCATGGAGAGAAGG
	Rev.	GGAGCAGCAAGCGATGAA
unc-3	For.	GAGAACTAAGCGCACCGAT
	Rev.	ATAACTGGAACTGAAGATGGCA
cha-1	For.	GCTGGAATTATTTAAGAAGGCTGTG
	Rev.	AGGGCGCAAAGATGATTGT
cho-1	For.	CCTTCCGTCAGATGTATCGTTT
	Rev.	GCTGTTGGTTCGGATGTACT

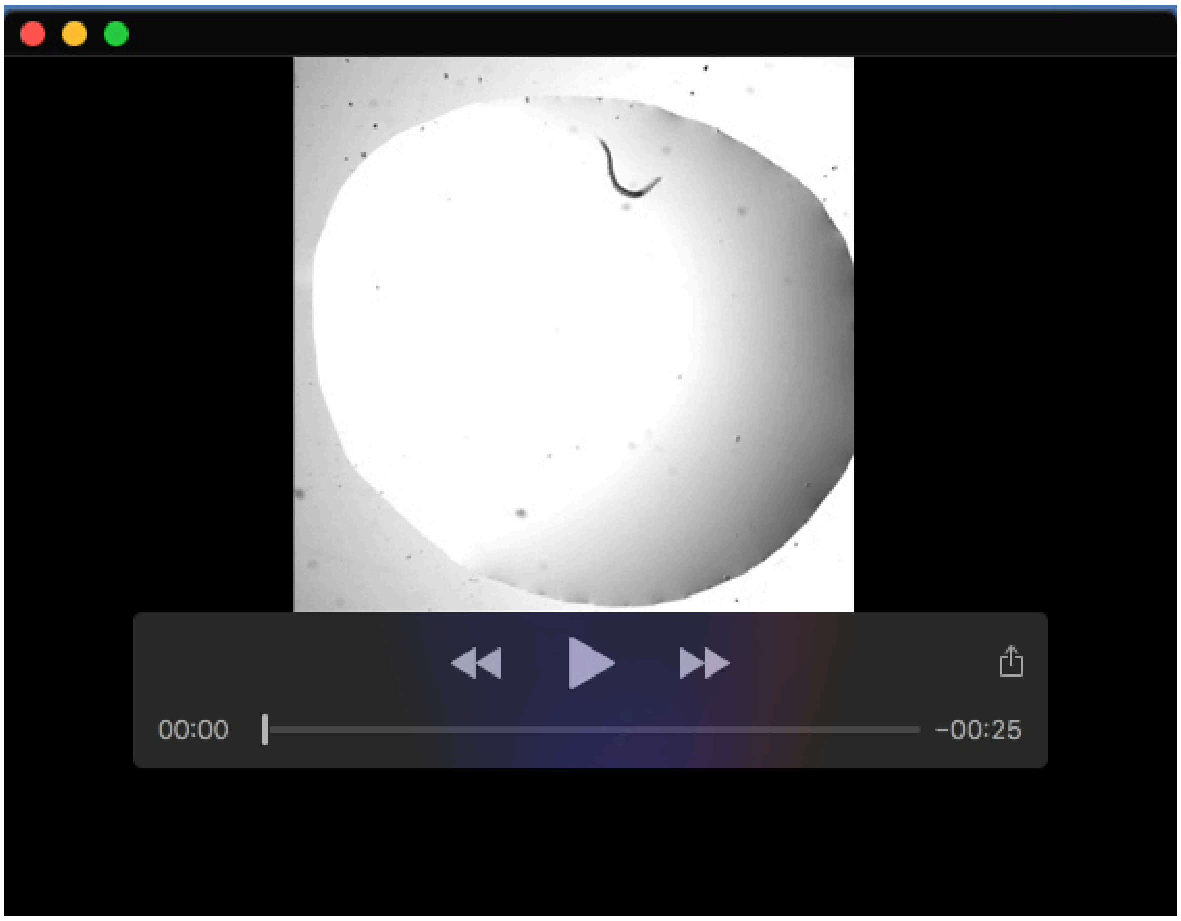

Movie 1. WT swimming analysis

Representative 10s video of WT animal swimming. Full analysis was of 60s videos.

Disease Models & Mechanisms • Supplementary information

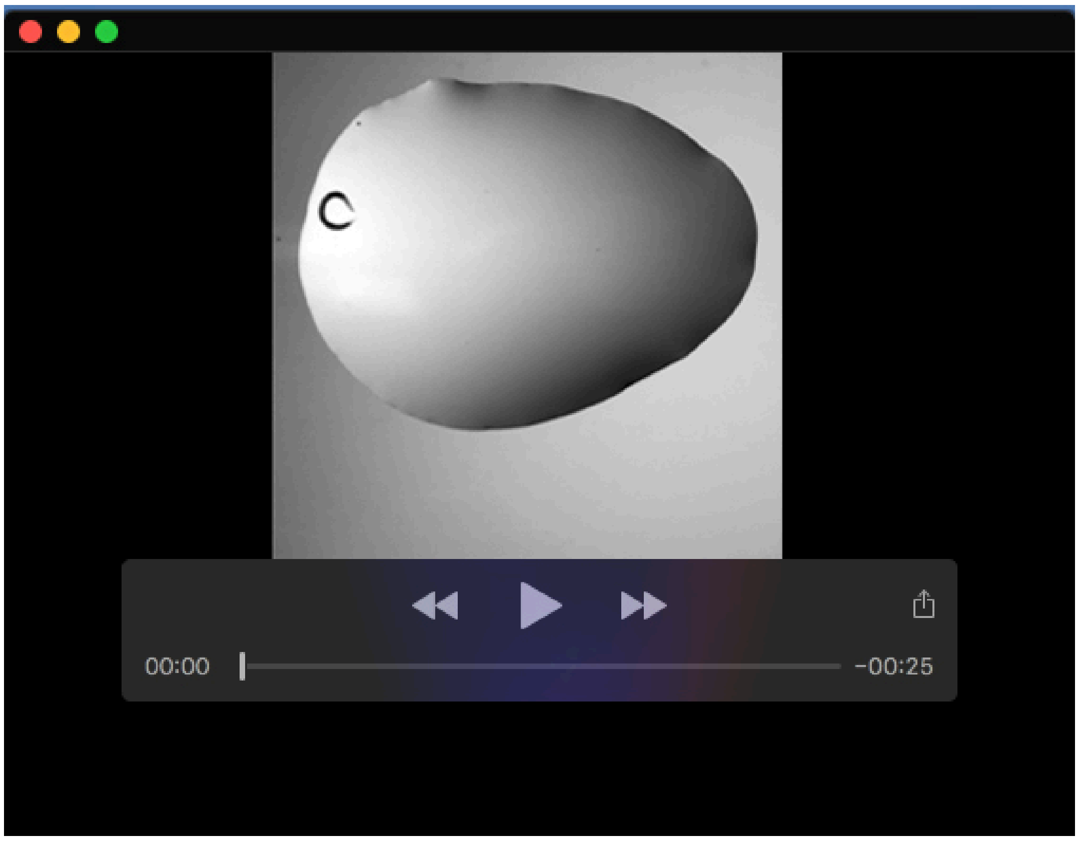

Movie 2. Gly147Arg swimming analysis

Representative 10s video of Gly147Arg animal swimming. Full analysis was of 60s videos.

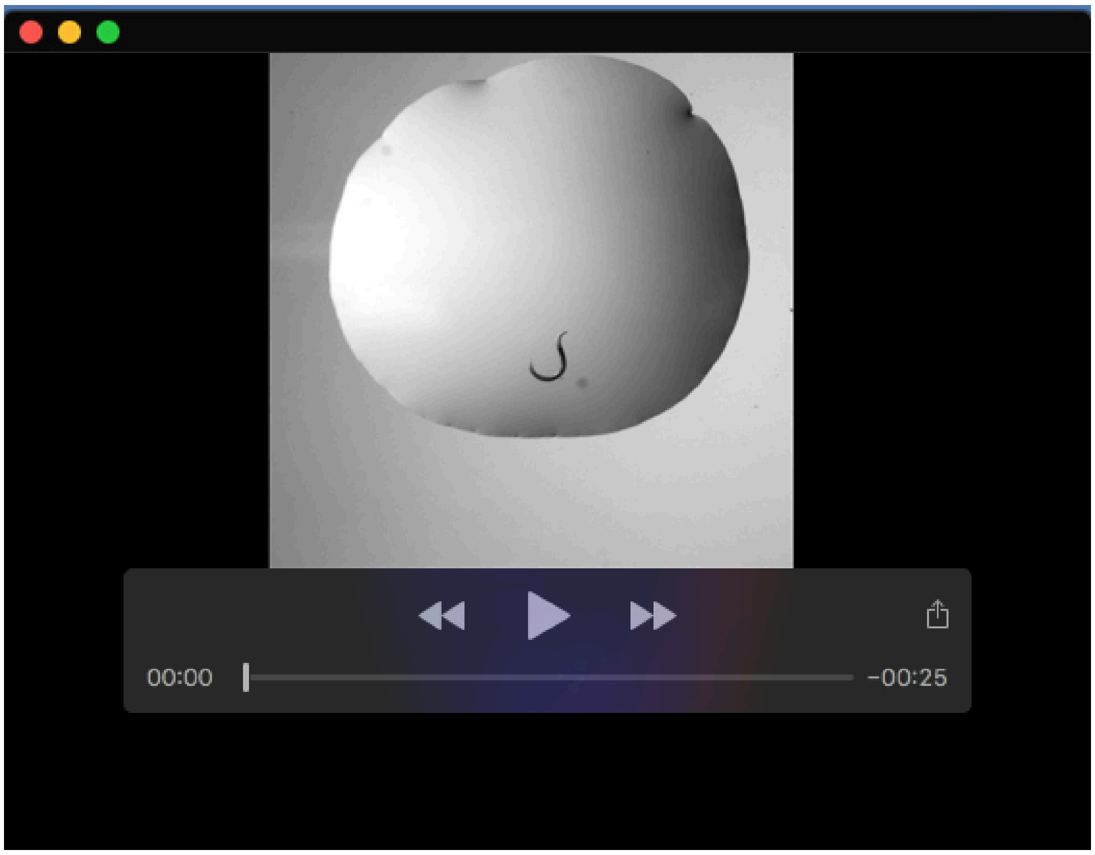

Movie 3. Gly166Cys swimming analysis

Representative 10s video of Gly166Cys animal swimming. Full analysis was of 60s videos.

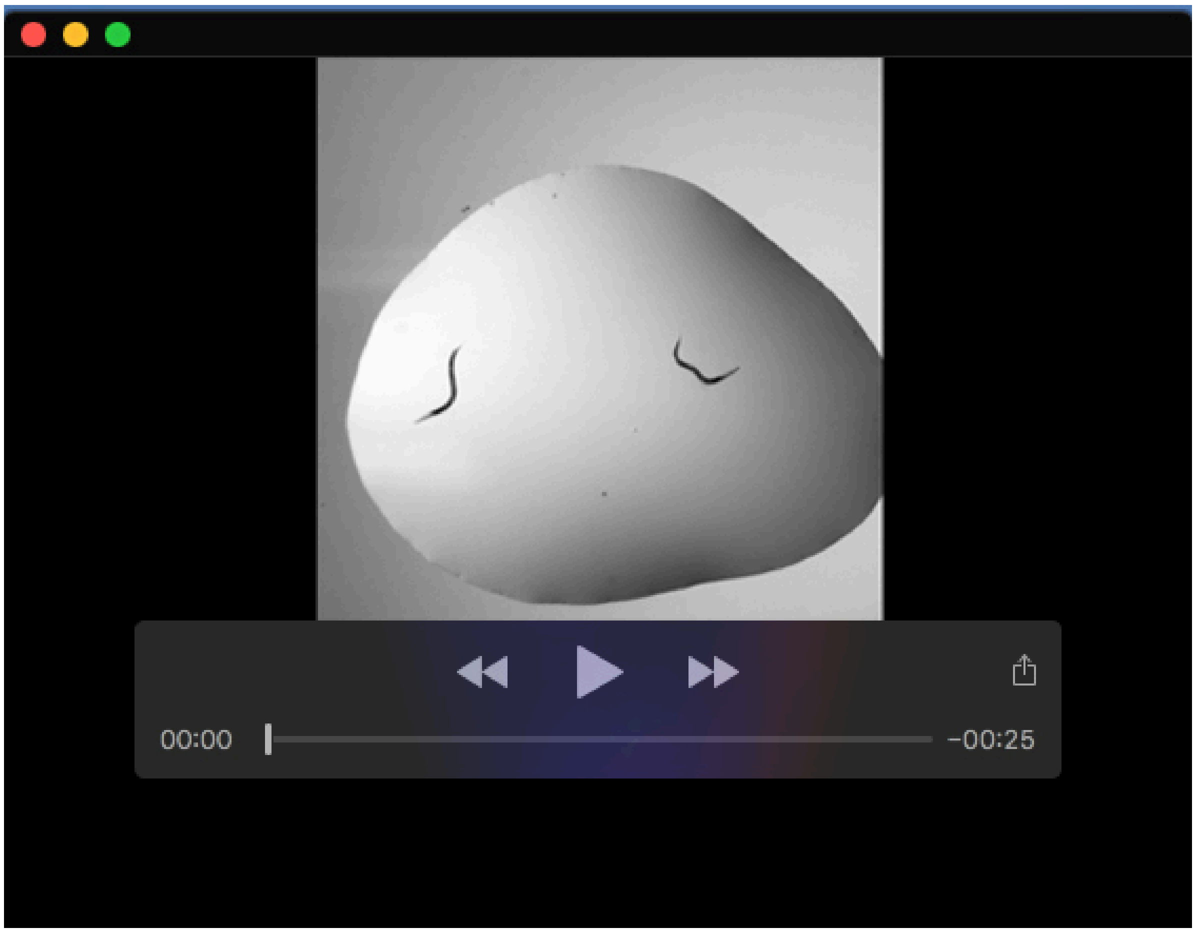

Movie 4. *nfu-1*Δ swimming analysis

Representative 10s video of *nfu-1*Δ animals swimming. Full analysis was of 60s videos.

Disease Models & Mechanisms • Supplementary information
